# Flavonoids: a natural remedy in the prevention and management of diverse diseases

**DOI:** 10.3389/fmed.2025.1713869

**Published:** 2025-11-17

**Authors:** Bo Yu, Jin Zhang, Mengxuan Zhu, Zongwu Li, Liqun Ren, Fan Zhang, Cuizhe Liu, Lin Zhang

**Affiliations:** 1 Hebei Key Laboratory of Nerve Injury and Repair, Hebei Province Key Laboratory of Research and Development for Chinese Medicine, Institute of Traditional Chinese Medicine, Chengde Medical University, Chengde, Hebei, China; 2 Hebei Tangwei Pharmaceutical Co., Ltd., Cangzhou, Hebei, China; 3 Chengde Medical University Library, Chengde Medical University, Chengde, Hebei, China

**Keywords:** flavonoids, pharmacological effects, antioxidation, anti-inflammation, anticancer

## Abstract

**Background:**

Flavonoids, which fall into the polyphenol family as secondary metabolites, can be widely found in traditional Chinese herbal medicines. Owing to their multi-target characteristics, low toxicity levels, and diverse sources, flavonoids have penetrated into assorted fields of contemporary medicine.

**Subjects and methods:**

We conducted a systematic search using databases including PubMed, ScienceDirect, Springer, Wiley, and Web of Science. The search employed keywords such as “flavonoids,” “heart,” “liver,” “lung,” “kidney,” “brain,” and “skin.” Studies on the therapeutic actions of diverse flavonoid compounds on diseases published between 2000 and 2025 were summarized and included in this review. Studies involving repeated flavonoid components that were published earlier, had poor relevance, or with unclear mechanisms of action were excluded. A total of 174 articles were finally selected.

**Results:**

The applications of flavonoids in addressing various health issues affecting the digestive, respiratory, integumentary, reproductive, endocrine, urinary, circulatory, and nervous systems highlights their significant role in systemic disease management.

**Conclusion:**

The application of traditional Chinese medicine has evolved from simple processing of raw medicinal materials to modern extraction and purification of active ingredients. Although aiming for precise therapeutic effects, acid/alkaline reagents or specialized technologies may disrupt the original structural integrity of these components. Combining the traditional theoretical essence with modern scientific techniques, we found that baicalin exists predominantly in the form of magnesium salt in *Scutellaria baicalensis* Georgi. This finding is expected to provide a reference for the development and utilization of effective components in traditional Chinese medicine.

## 1 Introduction

With the widespread global prevalence of cardiovascular ailments, cancer, neurodegenerative disorders, and metabolic syndromes, coupled with the continuous escalation of treatment costs, the global public health and medical system are facing increasingly considerable financial strain. The cumulative number of confirmed COVID-19 infections in recent years has surpassed 2 billion, further exposing vulnerabilities in global health infrastructures (1). Traditional infectious diseases such as malaria and dengue have seen a resurgence, driven by the impacts resulting from shifts in climate and the increasing resistance of pathogens to treatments (2, 3). The swift proliferation of newly identified infectious diseases (for instance, monkeypox) has further exacerbated public health crises, causing far-reaching impacts on the global economy, educational systems, and social order. These conditions are characterized by intricate pathophysiological processes and multiple contributing factors, with present therapeutic approaches predominantly centered around Western medicine. Nevertheless, treatments through Western medicine often focus on a narrow range of targets. Not only may its curative effect be limited, but it is also accompanied by multiple challenges such as low adherence among patients, significant rates of non-responsiveness to treatments, systemic adverse reactions, and substantial financial costs (4).

**FIGURE 1:**
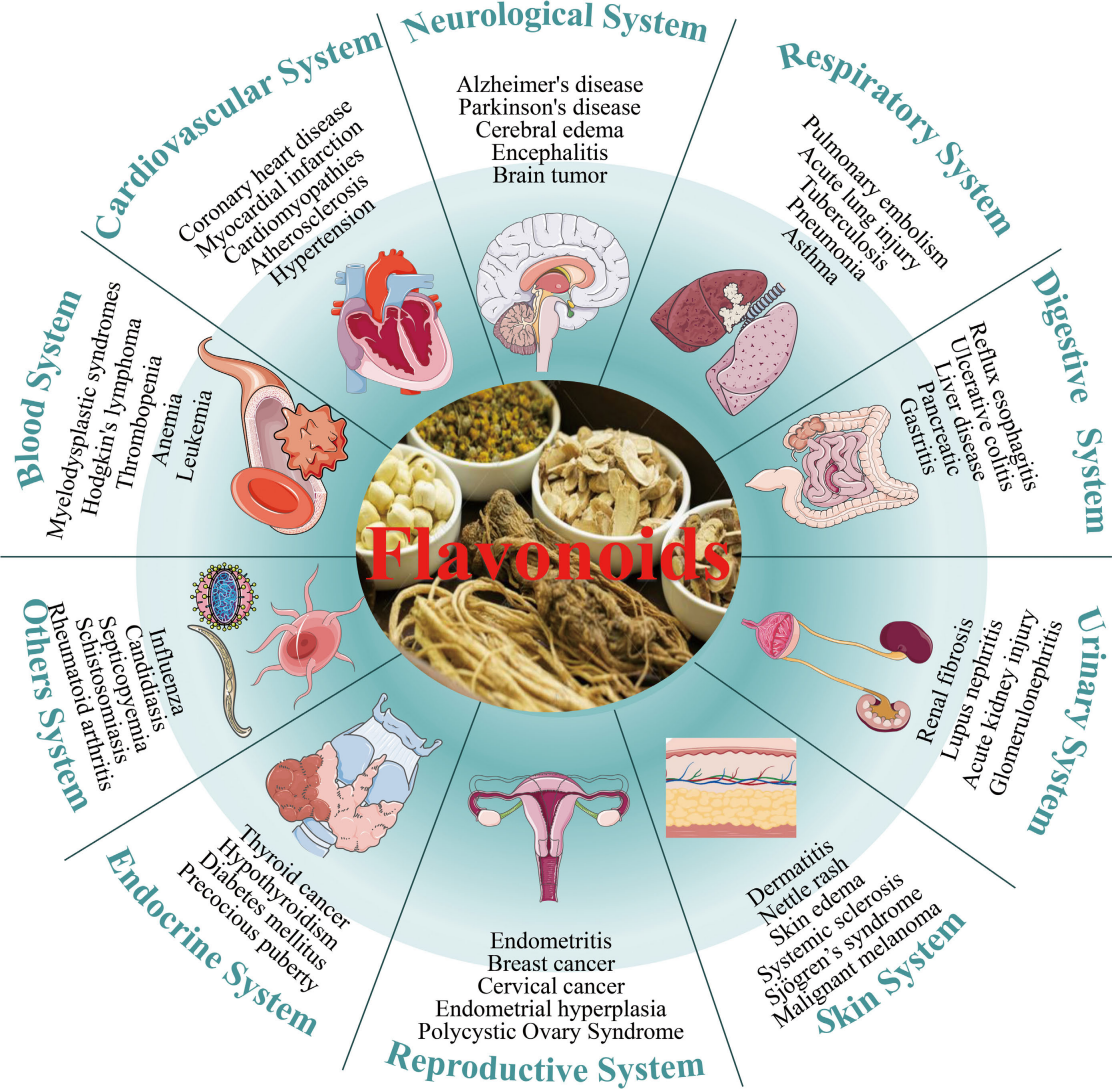
Numerous biological effects of flavonoids.

Recently, the effectiveness of traditional Chinese medicine in managing intricate health issues has become increasingly evident, leading to a rise in its acceptance and use. Chinese herbal medicines are distinguished by their multiple components, pathways, and target effects (5). Chinese herbal medicines encompass a diverse and intricate range of constituents, primarily consisting of compounds such as alkaloids, glycosides, steroids, polysaccharides, terpenoids, and others. Among them, flavonoids represent a significant group, essential for facilitating the therapeutic properties of Chinese medicine. Flavonoids constitute an important category of secondary metabolites found abundantly in numerous plants, including Chinese herbal medicines, fruits, vegetables (6). These compounds exhibit numerous biological effects (as illustrated in Figure 1), including anti-inflammation (7), anti-bacteria (8), anti-virus (9), anti-malaria (10), antioxidation (11), anti-angiogenesis (12), anti-cancer (13) and neuroprotection (14). Due to these diverse properties, they have found extensive applications in the clinical management of assorted diseases. Examples include silymarin capsules, baicalin tablets, wasabi capsules, troxerutin tablets, flavoxate hydrochloride tablets, and puerarin injection, among others (15, 16).

The efficacy of herbal medicines has been proven over centuries of extensive clinical application, which has traditionally relied on simple techniques such as decoction and grinding. However, as technological advances drive the modernization of herbal medicines and bring them in line with global standards, more attention is being paid to purifying and concentrating the active ingredients from the herbs rather than using the raw form directly. The main bioactive component of scutellaria baicalensis is baicalin, which is found in no < 9% of dried scutellaria, and even up to 20% in some samples. Our team found that the high content of baicalin in traditional scutellaria baicalensis decoction contradicts the documented water-insolubility. The current *Pharmacopoeia of the People’s Republic of China* (2025 edition) uses a water-extractable acid precipitation method to extract baicalin, which is claimed to increase its content from about 10% to more than 80%. However, this acid-induced process may disrupt its structural integrity and reduce its water solubility. Earlier studies showed that the appearance of scutellaria baicalensis decoction was clarified, and its baicalin concentration was about 44 mg/mL as determined by high-performance liquid chromatography (HPLC), which was much higher than the documented water solubility of baicalin (0.055 mg/mL) (17). However, acid and alkali reagents or special processes during purification may destroy the integrity of the constituents, resulting in a weakening of the pharmacological effects. This finding suggests that baicalin may exist in a form different from the currently known forms. Therefore, we hypothesized that a variant of baicalin with good water solubility might exist. Our research team successfully isolated baicalin magnesium (BA-Mg, whose structure consists of a magnesium ion bound to two baicalin molecules) from the traditional scutellaria baicalensis decoction by improving the extraction method and optimizing the process, and the related research has been granted invention patents in China, the United States of America, and the European Union (18). The discovery and application of BA-Mg represents an important breakthrough in the study of modern Chinese herbal medicine. Its innovative extraction process not only preserves magnesium ions, an important physiological active ingredient, but also significantly enhances water solubility and pharmacological activity. In addition, the research team developed a formulation process suitable for a variety of dosage forms, providing new ideas and strategies for the modern application of Chinese herbal medicine. These advances have enabled BA-Mg to demonstrate significant advantages in the treatment of various systemic diseases, laying a solid foundation for the modernized application of Chinese herbal medicine.

**FIGURE 2:**
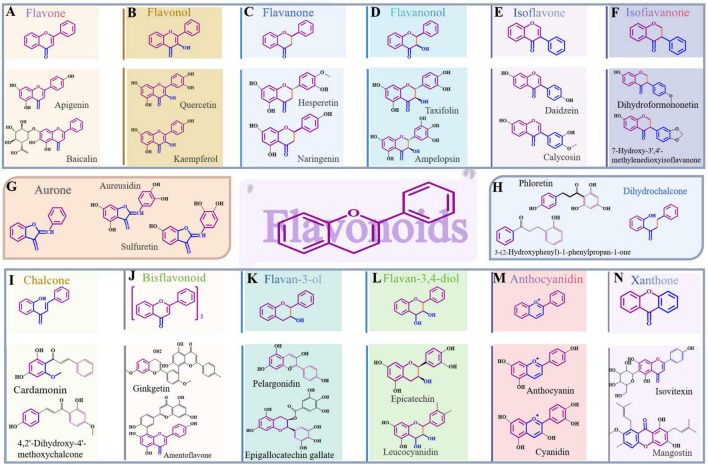
Structural categories of flavonoids alongside their representative compounds. **(A)** Flavone. **(B)** Flavonol. **(C)** Flavanone. **(D)** Flavanonol. **(E)** Isoflavone. **(F)** Isofiavanone. **(G)** Aurone. **(H)** Dihydrochalcone. **(I)** Chalcone. **(J)** Bisflavonoid. **(K)** Flavan-3-ol. **(L)** Flavan-3,4-diol. **(M)** Anthocyanidin. **(N)** Xanthone.

As diagrammed in Figure 2, flavonoids are primarily prompted by a parent core composed of 15 carbon atoms, which is created by linking two benzene rings (designated as ring A and ring B) via a three-carbon chain (referred to as the C-3 unit). This C-3 unit, along with a segment of ring A, combines to form an oxygen-containing heterocyclic structure known as ring C, which gives rise to a range of compounds characterized by a basic C6-C3-C6 carbon structure (19). Based on the oxidation level of the central three-carbon chain, the attachment site of ring B (either at the 2nd or 3rd position), and the presence or absence of ring formation in the three-carbon chain (20), these bioactive compounds, present in different plant parts, equip plants with a variety of medicinal properties and biological activities (21, 22). Flavonoids exhibit multiple structural types. In cases where a double bond exists between the 2nd and 3rd carbons of ring C and there is an absence of a hydroxyl group on the 3rd carbon, this is flavonoids, such as baicalin, apigenin, luteolin, and wogonin, etc. If there is a double bond between the 2nd and 3rd positions of ring C and a hydroxyl group at the 3rd position, they belong to flavonols, like quercetin, kaempferol, rutin, and fisetin. Flavonoids with a single bond between the 2nd and 3rd positions of ring C are flavanones, including typical examples like naringenin, hesperidin, chamaejasmine, and farrerol. When a single bond exists between the 2nd and 3rd positions of ring C and the 3rd position has a hydroxyl group, the corresponding flavonoids are flavanonols, including examples like dihydromyricetin, silibinin, silymarin, and astilbin. Flavonoids in which ring B is linked to ring C at the 3rd position are classified as isoflavones, such as glabridin, formononetin, prunetin, and calycosin. Compounds expressed by a single bond connecting the 2nd and 3rd positions of ring C, with ring B attached to the 3rd position, can be classified as isofiavanones, examples include dihydroformononetin, deguelin and sophoraisoflavanone G. Concomitantly, chalcones present an open ring C that forms a structure known as 1,3-diphenylpropenone, with typical representatives like cardamonin, isoliquiritigenin, and xanthohumol. In cases where a single bond connects the 2nd and 3rd positions of ring C, and ring B is linked to the 1st position of ring C, these compounds are categorized as dihydrochalcones, with notable representatives such as phloretin and phloridzin. Additionally, flavonoids that lack a double bond between the 2nd and 3rd positions of ring C but have a hydroxyl group at the 3rd position are categorized as flavan-3-ols, with this group encompassing compounds such as (-)-epigallocatechin gallate and procyanidin C1. If there is a single bond between the 2nd and 3rd positions of ring C and hydroxyl substitutions at both the 3rd and 4th positions, they are flavan-3,4-diol, e.g., (+)-epicatechin and catechin and among others. When ring C lacks hydroxyl substitution at the 3rd position, the carbonyl group at its 4th position is converted to a hydroxyl group, and the double bond between rings B and C undergoes a shift, the resulting flavonoids are anthocyanidins, including malvidin, cyanidin, and anthocyanin. Should ring C undergo a transformation to create a novel oxygen-inclusive heterocycle with ring A, forming a benzopyranone configuration, they are classified as a type of xanthones, such as garcinol and mangostin. Aurones represent a class of flavonoids marked by the relocation of a double bond in ring C shifts to the 3,4-position, while the linkage between rings A and C transforms from an α-pyrone configuration to a benzofuran arrangement, including compounds like bracteatin and coreopsin. As well, bisflavonoids are created through the connection of two flavonoid units via C-C or C-O-C bonds, including sotetsuflavone, ginkgetin, and kolaviron.

Given the rising incidence of diverse systemic illnesses and the limitations associated with current treatment modalities, it is imperative to explore alternative therapeutic strategies. Flavonoids, with their extensive biological activities and comparatively low toxicity, hold significant promise as complementary or even alternative therapeutic agents. The primary objective of this review is to provide a comprehensive and systematic overview of the mechanisms by which flavonoids function in the prevention and management of various systemic diseases. This study aims to elucidate the potential of flavonoids as innovative therapeutic agents, offering novel insights into the development of treatment methods centered around these compounds. By doing so, we hope to contribute to the development of innovative therapeutic strategies and improve global health outcomes, thereby addressing the urgent need for more effective and sustainable therapeutic options.

## 2 The therapeutic actions of flavonoids

### 2.1 Cardiovascular system

**FIGURE 3:**
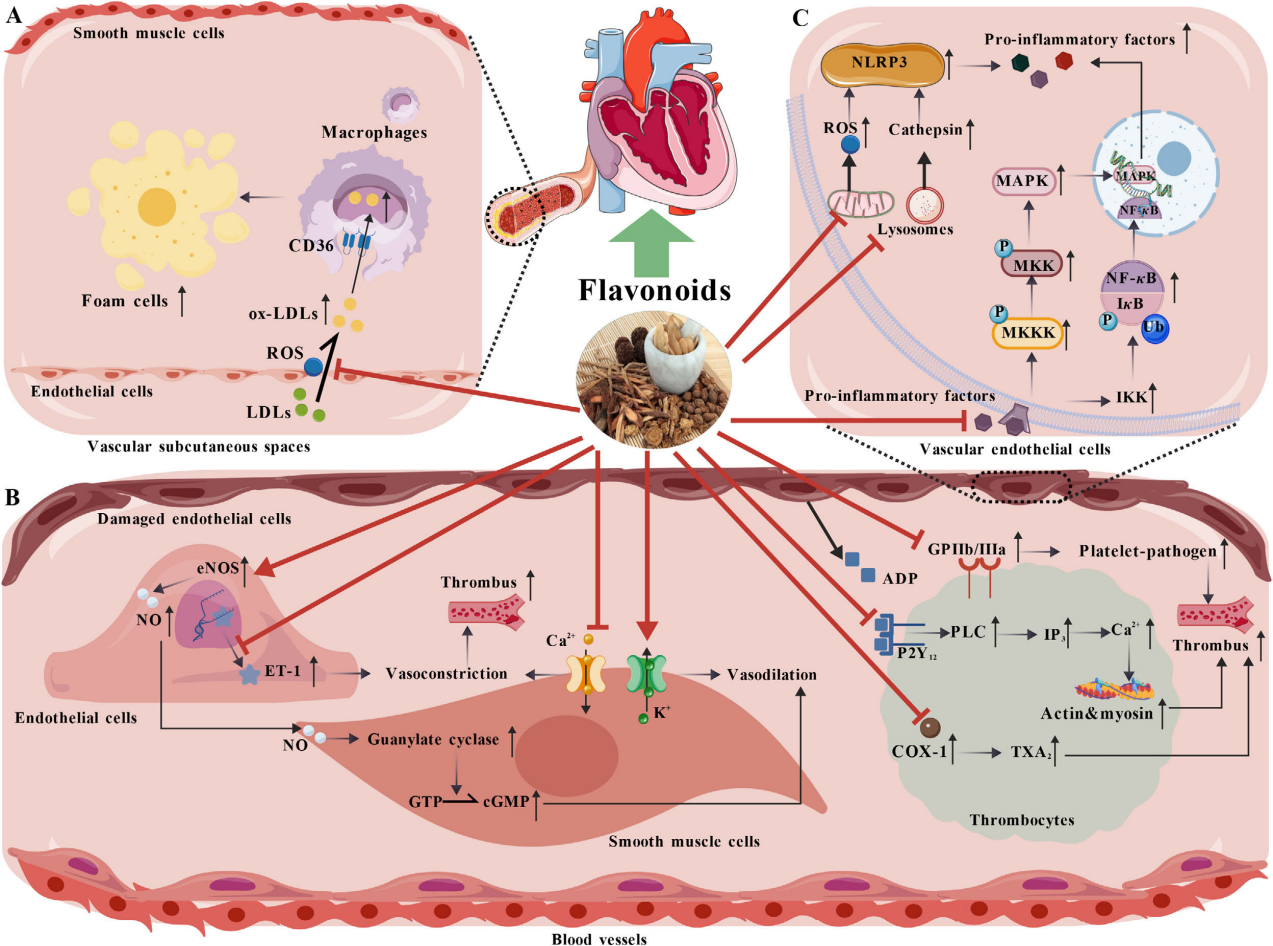
The mechanism of flavonoids in improving cardiovascular diseases. **(A)** Flavonoids exert antioxidant effects to prevent LDLs from oxidizing into ox-LDLs, limiting their infiltration into macrophages, inhibiting foam cell formation, and delaying atherosclerosis progression. **(B)** Flavonoids stimulate the eNOS pathway to promote vasodilation via NO, curb ET-1, regulate ion fluxes, block ADP-platelet binding, interfere with Ca^2 +^ signaling, inhibit COX-1 to reduce TXA_2_, and downregulate GPIIb/IIIa, thereby reducing platelet activation and aggregation to prevent thrombosis. **(C)** Flavonoids block NF-κB and MAPK activation in cardiomyocytes to reduce pro-inflammatory mediator release, attenuate mitochondrial ROS production and lysosomal cathepsin release, preventing NLRP3 assembly and further pro-inflammatory factor release, thus exerting anti-inflammatory effects.

The cardiovascular system functions to circulate blood through the body’s extensive network of blood vessels, ensuring that cells receive essential nutrients and oxygen while efficiently eliminating byproducts of cellular processes (as shown in Figure 3).

#### 2.1.1 Heart diseases

Coronary heart disease is a chronic lesion of the coronary arteries prompted by multiple factors. Corylin is a specific isoflavone isolated from *Psoralea corylifolia* L. The administration of corylin has been shown to engage Raf-1, conferring attenuation of the ASK1 activity. This interaction interrupts downstream apoptosis signaling pathways, which ultimately decreases the apoptosis of myocardial cells, hence enhancing cardiac performance (23).

Myocardial involvement can trigger focal or diffuse myocarditis. *Coxsackievirus* is one of the major pathogens of viral myocarditis. Icariin, a prenylated flavonol glycoside, reduced the viral load of *Coxsackievirus* 3 both in the cells and mice, prevented myocardial cell apoptosis by disrupting the S100 calcium binding protein A6/β-catenin/c-Myc signaling cascade, and alleviated *Coxsackievirus* 3-induced myocarditis by inhibiting proinflammatory cytokines (9).

Heart failure is characterized by the impairment of the heart’s ability to pump blood efficiently. Baicalin, a flavonoid, has the ability to mitigate the excessive stimulation of nicotinamide adenine dinucleotide phosphate hydrogen (NADPH) oxidase (NOX2), thus reducing oxidative stress, and offer notable protection to mice against the pathological modifications of the heart inflicted by isoproterenol, which involves the suppression of cardiac hypertrophy, improving the heart’s pumping capability, mitigating tissue scarring and alterations in heart structure, and managing chronic heart failure (24).

#### 2.1.2 Vascular diseases

Hypertension is a frequently encountered long-term condition stimulated by persistently high levels of arterial pressure. Quercetin, a naturally occurring flavonol, has been found to lower blood pressure in hypertensive rats that have been inflicted by N omega-Nitro-L-arginine methyl ester and improve pulse wave velocity β. Within renal arteries, quercetin reduces the heightened tension instigated by acetylcholine and enhances the contraction response triggered by phenylephrine. Regarding mesenteric arteries, quercetin causes concentration-dependent vasodilation and increases their sensitivity to acetylcholine-induced dilation, suggesting that it may help to reduce blood pressure by dilating resistance arteries (25).

A chronic disease, atherosclerosis arises from the thickening and hardening of arterial walls—this process leads to reduced flexibility and constriction of the lumen. Wogonoside, a flavone glycoside compound, led to a notable decrease in lipid deposition within the aorta, and alleviated the progression of atherosclerosis. Furthermore, wogonoside inhibited the secretion of the inflammatory marker levels in the aorta of ApoE^–/–^ mice, and decreased reactive oxygen species (ROS) levels, thereby mitigating the damage to vascular walls, safeguarding the functionality of vascular endothelial cells, diminishing lipid oxidation, and consequently impeded the development and advancement of atherosclerotic plaques (11).

Chronic venous insufficiency is frequently associated with inadequate return of venous blood. Diosmin, a natural flavone, is employed for addressing ailments of the vascular system. Research indicates that diosmin administration can diminish isoprostane levels in the plasma of patients suffering from chronic venous insufficiency by scavenging oxygen free radicals, hence mitigating oxidative stress-related harm and relieving the symptoms of chronic venous insufficiency. This particular benefit appears to be particularly notable in patients who smoke and suffer from chronic venous insufficiency (26).

An arteriovenous fistula refers to an abnormal channel between an artery and a vein, resulting in direct blood flow from the artery into the vein, bypassing the typical capillary system. Studies indicate that treatment with quercetin can mitigate kidney damage and vascular endothelial dysfunction in rats suffering from chronic renal failure, while by fostering the growth and reducing the cell death of endothelial cells from human umbilical veins when exposed to lipopolysaccharide (LPS) and serum derived from rats with chronic renal failure rats. Quercetin increases levels of NO as well as those of endothelial nitric oxide synthase (eNOS), upregulates the levels of phosphorylated eNOS expression, and promotes vasodilation and vascular endothelial protection. Moreover, quercetin downregulates the expression of erythropoietin-producing hepatocellular (EPH) receptor B4, EphrinB2, and p-caveolin-1, indicating that quercetin has the potential to effectively manage vascular health and prevent the failure of arteriovenous fistulas linked to chronic renal failure (27).

Deep venous thrombosis is influenced by the abnormal formation of clots within deep venous structures. Fisetin, a flavonol, notably blocks inflammatory signaling pathways mediated by MAPK and boosts the nuclear factor erythroid 2-related factor 2 (Nrf2) signal pathway, stimulating the production of various antioxidant enzymes to provide protective effects against oxidative damage on vessels and adjacent tissues, and thus treating deep venous thrombosis in mice inflicted by the surgical ligation of the inferior vena cava (28).

### 2.2 Respiratory system

#### 2.2.1 Nasal diseases

Nasal mucosa inflammation can result from viral infections, bacteria, allergens, etc. Cirsilineol, flavone compounds, reduces the levels of immunoglobulin E (IgE), prostaglandin D2, and leukotriene C4 (LTC4) in OVA-stimulated allergic rhinitis mouse. As well, cirsilineol treatment significantly decreases ROS and malondialdehyde (MDA) in smooth muscle cells challenged by OVA, and increases superoxide dismutase (SOD) levels, conferring improvement of allergic rhinitis symptoms (29).

Sinusitis is often driven by viruses, bacteria, or fungi. Luteolin is a natural flavone. It restores the maturity of olfactory marker protein and the immature state of growth associated protein 43 within olfactory sensory neurons. During this process, luteolin mitigates the apoptosis of olfactory sensory neurons by decreasing the amounts of cleaved caspase-3 and caspase-9 while enhancing the levels of the B-cell lymphoma-2 (Bcl-2) protein, which in turn alleviates symptoms of sinusitis (30).

Nasal polyps are benign proliferative diseases occurring in the nasal cavity. Eupatilin is a natural flavone compound. In a murine model of chronic rhinosinusitis manifested by nasal polyps established using OVA and staphylococcal enterotoxin B, eupatilin resulted in a notable decrease in polyp formation, epithelial thickness, and the thickness of the mucosal layer was lessened. Moreover, eupatilin exhibited the capacity to block epithelial-mesenchymal transition (EMT) by boosting E-cadherin levels and lowering the expression of N-cadherin (N-cad) and Vimentin in mice, which indicates that eupatilin could be a potential therapeutic choice for nasal polyp-associated chronic rhinosinusitis (31).

The nasopharyngeal region can give rise to a malignant tumor expressed as nasopharyngeal carcinoma. Isoquercitrin, a type of flavonol glycoside compound, diminished both the cell viability and the capacity for proliferation in CNE1 and HNE1 cell lines, which correlated with an increase in the ROS generation and lipid peroxidation, while suppressing the 5’-adenosine monophosphate (AMP)-activated protein kinase (AMPK) pathway. Isoquercitrin exerts an effect to impede the development of nasopharyngeal carcinoma (32).

#### 2.2.2 Throat diseases

Tonsillitis is a common upper respiratory tract disease primarily caused by diverse pathogens, notably bacteria and viruses. Vitexin, a flavone compound, was found to downregulate the toll-like receptor (TLR) 3 and TLR7 pathways in the mouse peritoneal macrophages infected with *influenza A virus* A/FM/1/47 (H1N1), hamper the synthesis of inflammatory mediator production, thus alleviating the symptoms associated with tonsillar inflammation (33).

Pharyngitis describes collectively referring to inflammation that arises due to microbial infections in the pharyngeal region. Studies indicate that dihydromyricetin, a naturally occurring dihydroflavonol, could downregulate the protein expression of inducible nitric oxide synthase (iNOS), TNF-α, and COX-2 in LPS-activated macrophages. Dihydromyricetin exerts its anti-inflammatory properties by inhibiting the phosphorylation of NF-κB, p38 kinase, and JNK (34).

#### 2.2.3 Tracheal and bronchial diseases

Bronchitis is an acute or chronic non-specific inflammation of the bronchial mucosa and adjacent tissues prompted by a variety of biological or non-biological factors. Myricetin, a flavonol compound, considerably inhibits the replication process of infectious bronchitis virus within chicken embryo kidney cells, while also increasing the transcriptional activity of NF-κB and interferon regulatory factor (IRF) 7 signaling cascade. By suppressing the deubiquitination activity of papain-like protease and boosting the ubiquitination modification levels of TNF receptor-associated factor 3 and 6, myricetin exerts a capacity to combat the infectious bronchitis virus effectively (35).

Asthma of the bronchial variety is a long-lasting pulmonary condition influenced by immune responses of the type 2 variety. Silibinin, a dihydroflavonol compound, alleviated allergic reactions prompted by house dust mite in asthmatic mice. This was accomplished through the suppression of NLR family CARD domain containing 4 (NLRC4) inflammasome activation and a decrease in the expression of matrix metalloproteinase-9 (MMP-9) in both house dust mite-exposed mice and stimulated human bronchial epithelial cell lines, suggesting its promise as a management option for asthma (36).

#### 2.2.4 Lung diseases

**FIGURE 4:**
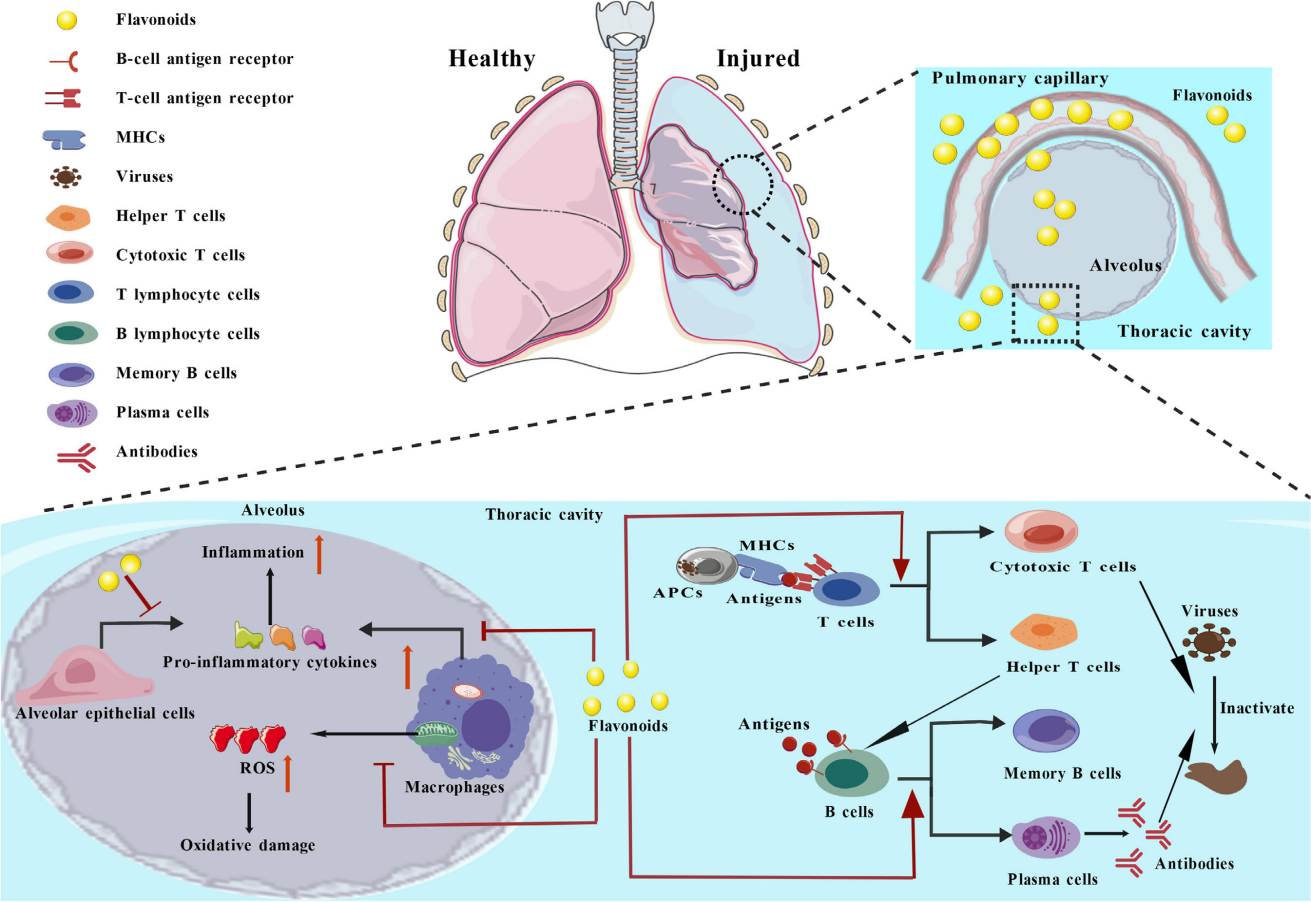
The role of flavonoids in the treatment of respiratory illnesses.

As depicted in Figure 4, pneumonia is a lung infection prompted by bacteria, virus, or various pathogens invading the lung parenchyma and proliferating in a way that surpasses the host’s defense capabilities. Such infections primarily target the alveoli, leading to exudates within the alveolar lumen and initiating cases of pneumonia. Hispidulin, a type of flavone present in assorted herbs, directly inhibited both the differentiation as well as proliferation of Th2 cells, leading to a decrease in the secretion of Th2-related cytokines. This mitigated hyperplasia of goblet cells in the airway, ultimately reducing allergic airway inflammation and type 2 lung inflammation (37).

Pulmonary fibrosis is marked by an increase in the number of fibroblasts and significant accumulation of extracellular matrix components. The findings indicated that alpinetin, a dihydroflavonoid, displayed therapeutic effects against pulmonary fibrosis through the inhibition of the transforming growth factor (TGF)-β/activin-like kinase 5 (ALK5)/Sma and Mad-related protein (Smad) signaling pathway, conferring attenuation of the transformation of fibroblasts into myofibroblasts (38).

Lung cancer is among the most common types of malignant neoplasms worldwide. Scutellarein belongs to the flavone class of compounds. Zhang et al. (13) indicated that scutellarein markedly suppressed the proliferation of lung cancer xenograft tumors. An important point to note is that, scutellarein significantly transformed the metabolism of amino acids, specifically impacting the metabolism of glutamine. It influenced essential transporters for glutamine, such as the alanine-serine-cysteine transporter 2 (ASCT2) and the L-type amino acid transporter 1, in addition to the activity of glutaminase GLS1, which subsequently led to a decrease in the levels of these proteins. Consequently, scutellarein demonstrates a significant capacity to suppress the proliferation of lung cancer by triggering cell death and reducing the metabolism of glutamine. Pulmonary hypertension can lead to alterations in the structural and function of pulmonary blood vessels. Oroxylin A, recognized as an effective flavone compound, could reduce the secretion of glucose transporters 1 (GLUT1), hexokinase 2, lactate dehydrogenase (LDH), and phosphoinositide-dependent protein kinase-1, alongside the enhancement of protein levels for both PK and isocitrate dehydrogenase 2, conferring remission of the average pulmonary artery pressure in a rat model for pulmonary arterial hypertension driven by monocrotaline (39).

Pulmonary emphysema is a chronic lung disease instigated by pathological states such as persistent abnormal air trapping, overinflation as well as destruction of the airway wall. Quercetogetin, a flavonol, suppressed the LPS-triggered activation of COX-2 and iNOS, reduced the synthesis of prostaglandin E2 (PGE2) and NO, suppressed IL-6, IL-1β, and TNF-α production, as well as lowering the nuclear translocation of NF-κB by disrupting the phosphorylation of its inhibitor in LPS-induced macrophage models. It furthermore inhibited inflammatory reactions via the extracellular regulated protein kinases signaling pathway, demonstrating its utility in treating pulmonary emphysema (40).

Acute respiratory distress syndrome presents as a severe, life-threatening condition of acute respiratory failure, marked by progressive dyspnea and refractory hypoxemia. Pomiferin, a compound belonging to the isoflavonoid class, could block the Akt/forkhead box protein O1 (FOXO1) signaling pathway, and suppress inflammation and oxidative harm, showing potential benefits for acute respiratory distress syndrome (41).

#### 2.2.5 Pleural diseases

Pleurisy refers to the swelling that takes place within the pleural cavity due to infections triggered by bacteria, viruses, fungi, or other pathogens. Amentoflavone is a bisflavonoid analog found in the peppermint plant. Hou et al. (42) established a model of carrageenan-induced pleurisy in mice and found that amentoflavone effectively attenuated pleural effusion and inflammatory response by blocking pro-inflammatory signaling pathways including NF-κB, transcription (STAT)3, as well as extracellular signal-regulated kinase 1/2 (ERK1/2). Amentoflavone activates Nrf2 by dissociating Kelch-like epichlorohydrin (ECH)-associated protein 1 (Keap1), subsequently elevating the concentrations of heme oxygenase-1 (HO-1), NAD(P)H-quinone oxidoreductase 1 (NQO1) and γ-glutamylcysteine ligase, which act as an anti-inflammatory and antioxidant, ultimately improving pleurisy and pleural effusion.

### 2.3 Digestive system

**FIGURE 5:**
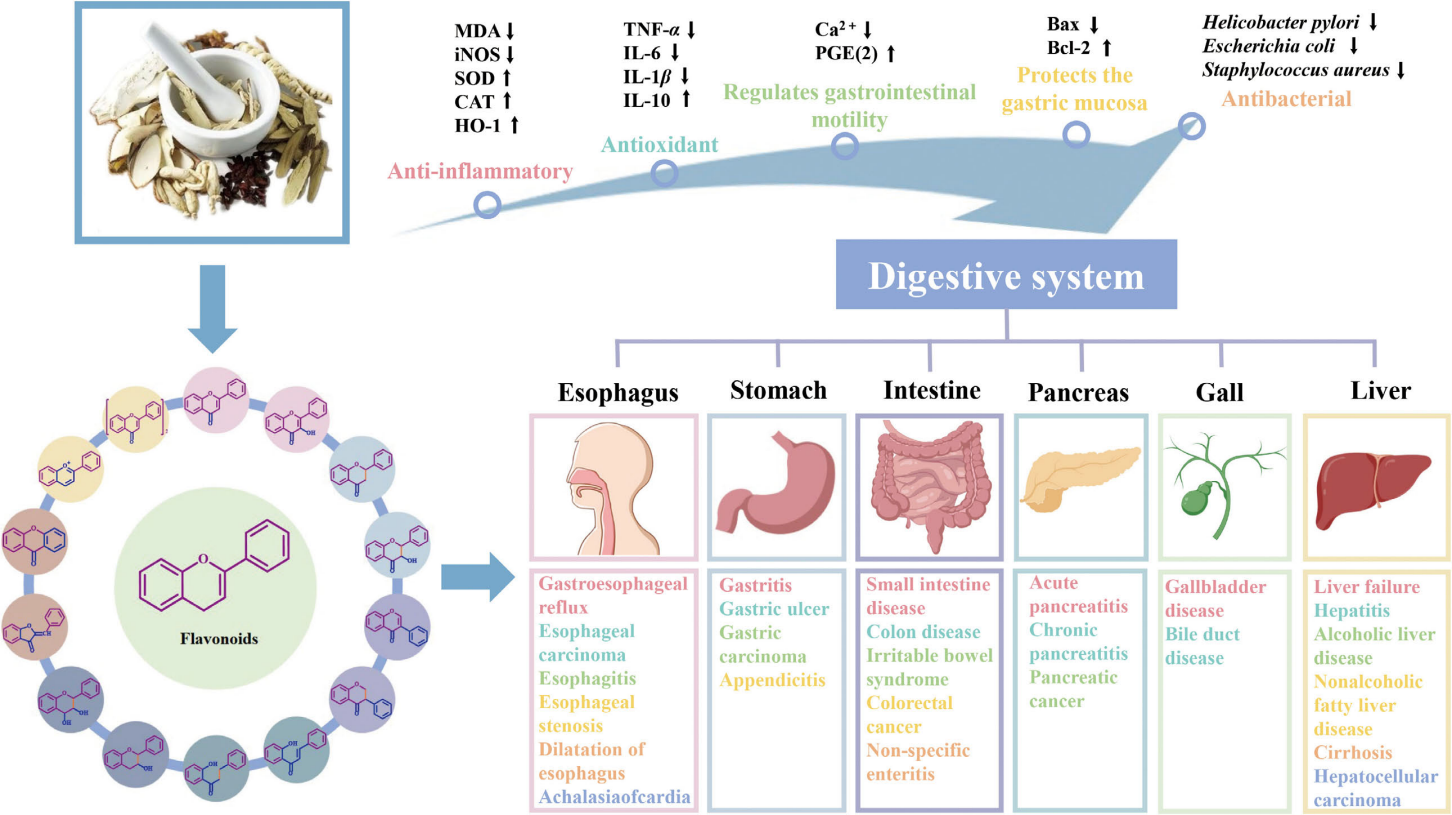
Mechanism of action of flavonoids on the digestive system and related ailments.

Disorders affecting the digestive system are not uncommon, often involving major lesions within the esophagus, stomach, liver, gallbladder, pancreas and intestines (as indicated in Figure 5).

#### 2.3.1 Esophageal diseases

Eosinophilic esophagitis is instigated by the presence of eosinophils and leading to esophageal dysfunction, which may also present with signs such as regurgitation, feeding difficulties, and stomach discomfort. The flavone compound 7,4’-dihydroxyflavone exhibits considerable anti-inflammatory and immunomodulatory effects through the suppression of eosinophil attractants such as CCL26, Th2 cytokines, including IL-4, IL-5, and IL-13, along with particular types of IgE. In a culture system derived from human esophageal biopsy, 7,4’-dihydroxyflavone markedly diminished the levels of inflammatory mediators and associated genes, by inhibiting upstream NF-κB signaling pathways, and thus alleviated the eosinophilic esophagitis (43).

Esophageal squamous cell carcinoma (ESCC)—the main type of esophageal carcinoma (EC)—is a severe malignant tumor in the digestive system. 5,7,4’-trimethoxyflavone, a variant of flavone, is able to bind to leucine-rich PPR motif-containing proteins, STAT3, and cyclin dependent kinase (CDK) 1, which subsequently disintegrates the associations of LRPPRC-janus kinase (JAK)2-STAT3 and JAK2-STAT3-CDK1, thereby hindering the progression of ESCC by disrupting the associated protein complexes, thereby preventing tumorigenesis and inhibiting tumor growth (44).

#### 2.3.2 Gastric diseases

Vitexin treatment notably reduces the generation of pro-inflammatory cytokines, while also attenuating the activity of the NOD-like receptor protein 3 (NLRP3). Additionally, it notably relieved 1-methyl-3-nitro-1- nitrosoguanidine-induced chronic atrophic gastritis in rats, as it prevented body weight loss and reduced gastric tissue damage (45).

Zapotin, a polymethoxylated flavonoid, activated the apoptosis pathway with mitochondria, up-regulating the expression and activity of apoptosis-associated proteins, thus encouraging apoptosis in the gastric carcinoma cell line SNU-1. Additionally, zapotin hinders the proliferation of gastric cancer cells by suppressing the expression and activity of key proteins linked to the m-TOR/PI3K/Akt signaling pathway, including PI3K, Akt, and m-TOR (46).

#### 2.3.3 Liver diseases

Acute liver failure is a distinctive clinical syndrome with a high risk of mortality. Naringin belongs to the class of dihydroflavonoid glycosides. Research has indicated that the prophylactic administration of naringin alleviated liver damage driven by acetaminophen via enhancing the level of antioxidative enzymes, inhibiting the production of inflammatory mediators and engaging the apoptotic pathway. Notably, naringin can act as a key enhancer of cation transport regulator-like protein 2 (CHAC2), thus safeguarding mice against acetaminophen-induced acute liver injury by activating the Nrf2 pathway mediated by this protein (47).

Viral hepatitis refers to a group of infectious diseases predominantly liver lesions arised from a variety of hepatitis viruses. Dihydromyricetin showed a marked reduction in both hepatitis B virus (HBV) replication and the release of HBsAg and HBeAg. Concurrently, dihydromyricetin inhibited the expression levels of HBV-related mRNA. Concomitantly, dihydromyricetin lowered the mRNA expression of HNF4α, a change that then suppressed HBV replication (48).

Excessive or long-term consumption of alcoholic beverages can cause alcoholic-associated liver disease. Iron overload is a common characteristic of alcoholic liver disease. Oligomeric proanthocyanidins are a type of polyphenol that falls under the flavan-3-ol category. The presence of oligomeric proanthocyanidins has been shown to decrease the release of cathepsin B (CTSB), which in turn mitigates the rise in pyroptosis-associated proteins triggered, including proteins involved in the NLRP3, cleaved-caspase 1, and IL-18, this subsequently attenuating acute alcoholic liver injury (49).

Metabolic dysfunction-associated steatotic liver disease (MASLD) includes a spectrum of conditions comprising steatohepatitis, as well as its associated fibrosis and cirrhosis. Hesperetin is a naturally occurring dihydroflavone. A study indicated that hesperetin significantly reduced body weight, improved lipid metabolism disorders and mitochondrial dysfunction in rats with Non-Alcoholic Fatty Liver Disease (NAFLD). Hesperetin reduced dynamin-related protein 1 (Drp1) expression, heightened the expression of mitochondrial fusion proteins known as mitofusin-2 and optic atrophy 1, while simultaneously suppressing the activity of fission protein 1 and preventing the progression of NAFLD (50). As well, puerarin is a member of the isoflavone category. In addition, puerarin enhanced the expression of p-ULK1 in these immune cells, which activated autophagy and decreased plasminogen activator inhibitor-1 (PAI-1) levels. Puerarin adjusted the polarization profiles of macrophages by activating the STAT3/hypoxia-inducible factor (HIF)-1α and PI3K/Akt signaling pathways, while reducing the accumulation of hepatic lipid droplets, the count of cytoplasmic vesicles that reduces structural liver irregularities and mitigates non-alcoholic steatohepatitis (51). The researchers found that eupatilin could inhibit the β-catenin/PAI-1 signaling pathway, confer mitigation of the expression of fibrosis markers COL1α1 and α-SMA, along with the EMT marker N-cadherin, consequently alleviating liver fibrosis (52).

Hepatocellular carcinoma is viewed as one of the most prevalent types of malignant neoplasms. Prunetrin, which falls under the category of isoflavones, has been found to promote arrest at the G2/M checkpoint by down-regulating critical cell cycle regulators, including CDK1/2 and cyclin B1. Concomitantly, prunetrin exerts its capability against hepatocellular carcinoma by stimulating the MAPK pathway through elevated phosphorylation levels of both p38 and ERK (53).

#### 2.3.4 Biliary tract diseases

Luteolin modulates the immune response by inhibiting the antigen-presentation pathways related to major histocompatibility complex (MHC) class I and II which are highly activated in both high-fat diet (HFD) and ARE-Del-/- mice. Additionally, it strongly stimulates peroxisome proliferator-activated receptor (PPAR) signaling, which is diminished in mice, exerting a therapeutic effect on primary biliary cholangitis (54).

Schistosomiasis is a chronic parasitic infection affecting the liver and intestines. Licochalcone B is a class of substances known as chalcones. The administration of licochalcone B causes complete mortality among mature *Schistosoma mansoni*, and dramatically decreases their egg-laying and locomotor activity, while it has no effect on mammalian Vero cells. Simultaneously, licochalcone B induces alterations in the morphology of the ectoderm in *Schistosoma mansoni* worms, with a dose-related effect on the quantity of nodules present. Research has indicated that licochalcone B was shown to be effective against schistosomiasis by inhibiting the activity of ATP bisphosphate hydrolase and adenosine diphosphatase in *Schistosoma mansoni* organisms (55).

#### 2.3.5 Pancreatic diseases

Tectoridin, a type of isoflavone, significantly attenuated the pancreatic injury, reducing the serum levels of amylase and lipase. Tectoridin also encourages macrophage polarization toward the M2 subtype, attenuate the severity of inflammation, promote organizational repair, thus treating severe acute pancreatitis (56). Dihydrokaempferol is a natural dihydroflavanol extracted from Bauhinia championii, was able to reduce Keap1 levels while enhancing the transcriptional activity of nuclear Nrf2, which promotes the expression of antioxidation-related genes and exerts antioxidant activities, ameliorating the damage to the pancreas instigated by acute pancreatitis (57).

#### 2.3.6 Intestinal diseases

Ulcerative colitis (UC) is characterized by mucosal inflammation starting distally, which may extend proximally and even involve the entire colon. Genkwanin, a flavone, inhibited ROS production, upregulated sirtuin 1 expression, and improved mitochondrial function, including an elevation in the rate of oxygen utilization, heightened levels of mitochondrial DNA, along with enhanced functioning of the electron transfer chain complex I, II, and IV activity, contributing to the reduction of ulcerative colitis (58).

Irritable bowel syndrome (IBS) is a functional intestinal condition typically instigated by visceral hypersensitivity and gut microbiota dysbiosis, leading to abnormal bowel-brain interactions. From one side, luteolin acts to hamper the proliferation of *Helicobacter pylori* and modulate the activity of arylamine N-acetyltransferase by decreasing the acetylation degree of 2-aminofluorene or p-aminobenzoic acid, which is significant in addressing duodenal ulcers (59).

As the third most prevalent cancer worldwide, colorectal cancer (CRC) is recognized as one of the most lethal malignancies globally (60). Cudraflavone C, a type of flavonoid, has the capacity to hamper the expression of downstream pro-oncogenes by inhibiting either the GPX2 expression or activity, blocking GPX2-dependent activation of the Wnt/β-catenin pathway, inhibiting downstream pro-carcinogenic gene expression, thus demonstrating a shielding effect against CRC (61).

### 2.4 Hematological system

The hematological system along with its associated organs, which have the functions of transporting oxygen, nutrients and metabolic wastes, while regulating body temperature and acid-base balance, as well as participating in immune defense and coagulation, which are essential for sustaining essential functions of the human body.

#### 2.4.1 Disorders of red blood cells

Anemia is driven by a reduction in the usual quantity of red blood cells or concentration of hemoglobin within them. Chrysin, a naturally occurring flavone, reduced the serum activity of liver enzymes associated with hepatocellular damage, thereby ameliorating iron-overload- activated hepatocyte damage. What’s more, chrysin markedly up-regulated the inflammation-controlling proteins. It also enhanced hepatic iron loading and activated NLRP3, while inhibiting the acetylation of NF-κB and its movement into the nucleus. Simultaneously, the application of chrysin resulted in increased Bcl-2 protein levels, reduced the levels of pro-apoptotic protein Bax (linked to Bcl-2) as well as caspase-3 activity, and effectively prevented iron overload-triggered apoptosis (62).

#### 2.4.2 Disorders of white blood cells

Acute lymphocytic leukemia is characterized by genetic alterations and chromosomal irregularities, which contribute to the abnormal proliferation and impaired differentiation of lymphoid precursor cells. In acute lymphocytic leukemia cells, apigenin, a flavone compound, substantially diminished Bcl-2 protein levels while enhancing the levels of Bax, along with activating cleaved caspase-3 and -9, ultimately leading to apoptosis. The administration of apigenin notably enhanced the levels of phosphorylated AMPK as well as SIRT1 in SUP-B15 and Jurkat cell lines, suggesting that apigenin has a pro-apoptotic effect on acute lymphocytic leukemia cells (63).

Hodgkin’s lymphoma, formerly referred to as Hodgkin’s disease, is an uncommon type of monoclonal lymphoid tumor. Non-Hodgkin lymphoma represents a neoplastic condition impacting lymphocytes, often initiating in the lymphatic system. Flavokawain B, a chalcone analog, is active by disrupting the MMP, activating cleavage of PARP, thereby triggering mitochondrial apoptosis to inhibit the proliferation of non-Hodgkin’s lymphoma SUDHL-4 cells (64).

#### 2.4.3 Platelet disorders

An abnormal decrease in the quantity of platelets in the bloodstream is a manifestation of thrombopenia. Genistin is one of the active ingredients in soy isoflavones, which has estrogen-like effects. Genistin enhanced the transcriptional activity of nuclear estrogen receptor beta (ERβ) through activation of membrane receptor-mediated PI3K/Akt and MEK/ERK signaling pathways, and promoted megakaryocyte differentiation and thrombopoiesis through direct binding to ERβ, hence ameliorating X-ray-caused thrombocytopenia in mice (65).

Disseminated intravascular coagulation is a syndrome acquired through the widely distributed microvascular thromboses coupled with a simultaneous depletion of both blood platelets and coagulation factors. Myricetin-treated inhibited platelet granule release when stimulated with thrombin, resulting in a reduction of P-selectin levels. Simultaneously, myricetin notably impaired the activation of thrombin- triggered platelet integrin and blocked the signaling pathway associated with integrins, which subsequently diminished platelet adherence, spreading and clot retraction, and blocked thrombus formation. As well, myricetin exhibited a notable decline in the number of platelets adhering to surfaces and a reduction in the area over which these platelets spread, reducing the degree of platelet aggregation and improving coagulation (66).

### 2.5 Urinary system

**FIGURE 6:**
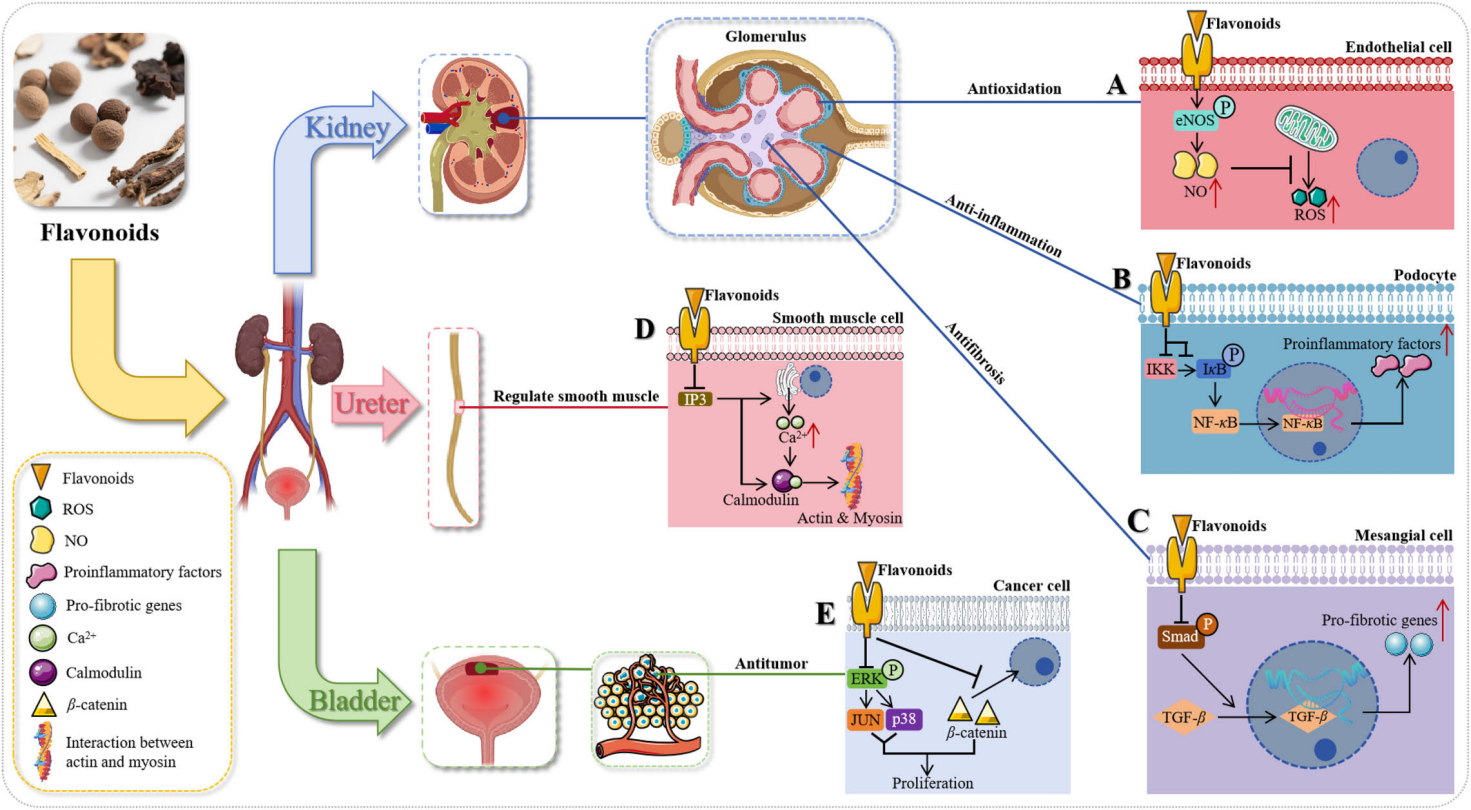
The role of flavonoids in addressing diseases related to the urinary system. **(A)** In endothelial cells, flavonoids activate eNOS phosphorylation to enhance NO production and counteract ROS, exerting antioxidant effects. ** (B)** In podocytes, flavonoids obstruct IKK-mediated IκB phosphorylation, inhibit NF-κB activation, and reduce pro-inflammatory agent release, playing anti-inflammatory roles. ** (C)** In mesangial cells, flavonoids inhibit TGF-β -Smad binding and activation, reducing pro-fibrotic gene expression to exert anti-fibrotic effects and improve renal fibrosis. ** (D)** In smooth muscle cells, flavonoids mitigate the IP3 signaling pathway, reducing Ca^2 +^ release and its interaction with calmodulin, weakening actin-myosin coupling to enhance ureteral smooth muscle function and exert diuretic effects. ** (E)** In cancerous cells, flavonoids activate the ERK signaling pathway, leading to phosphorylation of JUN and p38 MAPK, upregulating β-catenin and promoting malignant cell apoptosis, exerting anti-cancer properties.

Diseases of the urinary system primarily present in the urinary tract with symptoms like alterations in urination, lumps, and pain, but they may also manifest through various symptoms elsewhere, including hypertension, edema, and anemia (as reflected in Figure 6).

#### 2.5.1 Kidney disease

Kidney failure is defined as a syndrome of decreased or severely compromised renal functionality, leading to diverse metabolic imbalances. Kaempferitrin is a type of flavonol extracted from the foliage of *Zingiber officinale*. Kaempferitrin reduced iron, 4-hydroxynonenal, and MDA concentrations while enhancing GSH levels, and mitigated NOX4-mediated iron mutations in renal tubular cells, offering potential therapeutic value for chronic renal failure (67).

Fibrosis of the kidneys is a frequent mechanism associated with the progression of chronic renal disease. Morin hydrate, a flavonol, managed ionizing radiation-induced acute kidney injury in rats, prevented nephritis and renal fibrosis by enhancing the SIRT1/Nrf2/miR-125b pathway while promoting the production of various antioxidant enzymes (68).

Renal cell carcinoma represents a notably aggressive malignancy within the urinary system. Epigallocatechin-3-gallate, a flavan-3-ol compound, hinders both the migration and invasion of the human renal cell carcinoma cell lines like 786-O cells and ACHN cells, as well as EMT. This occurs through the activation of the autophagy pathway facilitated by the transcription factor EB, thus treating renal cell carcinoma (69).

Severe infection, created by pathogenic bacteria, fungi, viruses and parasites, could lead to a systemic response instigated by widespread inflammation, known as sepsis. Cynaroside, flavonoids, reinstated the functioning of pyruvate kinase and curtailed the action of proteins tied to glycolysis, while also impacting the HMGB1 hyperacetylation linked to glycolysis in septic livers (70).

Nephrotic syndrome is prompted by dysfunctional glomerular filtration disorders. Procyanidin B2, a form of flavan-3-ol, has been identified. Treatment with procyanidin B2 has demonstrated therapeutic effects on nephrotic syndrome by counteracting oxidative stress and dampening inflammatory reactions, improving podocyte cell death and dysfunction in autophagic processes, as well as reducing the electrolyte imbalances and swelling (71).

The development of polycystic kidney is significantly influenced by genetic abnormalities that lead to altered cellular proliferation and an atypical formation of the mesenchymal stroma. Panduratin A, a type of chalcone, has shown to diminish collagen deposition within renal tissues, notably the cystic index, and has therapeutic properties for polycystic kidney (72).

Renal artery stenosis is categorized as a vascular disorder impacting the kidneys, frequently driven by atherosclerosis or fibromuscular dysplasia. Galangin, a flavonol, curtailed tissue markers of oxidative stress while enhancing catalase (CAT) activity, which subsequently contributed to the mitigation of hypertension inflicted by renal artery stenosis, alongside the associated cardiac and renal injuries in rats (73).

#### 2.5.2 Bladder disease

Cystitis is an inflammatory condition that occurs in the bladder, which is primarily instigated by both specific and non-specific bacterial infections. Quercetin has been shown to enhance the expression of lipoprotein lipase, enhance the production of glycosaminoglycan while decreasing myeloperoxidase (MPO), IL-1β, and TNF-α levels, so as to attenuate bladder injury and mast cell degranulation in a rat model of interstitial cystitis/bladder pain syndrome (74).

The incidence rate of bladder tumors ranks 2nd only to prostate cancer among male urogenital tumors. The introduction of orientin applied to T24 human transitional cell bladder carcinoma cells significantly decreased the levels of shh, p-smo, smo and Gli2 associated with the hedgehog signaling pathway, along with decreasing the expression of p-p65, which serves as an activator for the NF-κB signaling cascade, suppressed the growth of cells while enhancing cell apoptosis (75).

### 2.6 Reproductive system

#### 2.6.1 Prostatosis

A common urological disease among the elderly is benign prostatic hyperplasia. Kolaviron, a bisflavonoid, alleviates the prostate weight by inhibiting the production of 5-α reductase, dihydrotestosterone, as well as androgen receptor. In furtherance, kolaviron alleviates oxidative damage by boosting the functionality of antioxidant enzymes. Additionally, it mitigates the inflammatory reactions through the suppression of inflammatory mediators. Besides that, kolaviron inhibits oxidative stress by lowering the expressions of Ki-67 antigen, vascular endothelial growth factor (VEGF), and fibroblast growth factor. Kolaviron also promotes apoptosis and addresses benign prostatic hyperplasia by decreasing Bcl-2 levels while enhancing the activities of tumor suppressor proteins p53 and caspase-3 (76).

Prostate cancer is acknowledged as one of the most widespread forms of cancer worldwide. Gambogenic acid, a compound belonging to the flavone class, has been found to trigger a halt in the cell cycle at the G2/M and S stages in both PC-3 and DU145 human prostate cancer cells, resulting in the attenuation of cell proliferation. Concomitantly, introduction of gambogenic acid reduces MMP, leading to the initiation of apoptosis in PC-3 and DU145 cell lines. Additionally, gambogenic acid has the potential to instigate stress within the endoplasmic reticulum, initiate the signaling pathway involving JNK, as well as elevate the phosphorylation states of JNK and Jun proteins in cells, effectively promoting both autophagy and apoptosis in PC-3 cell lines (77).

#### 2.6.2 Diseases of other organs

Testicular cancer is a rare malignant tumor. El-Diasty et al. (78) induced testicular cancer in rats using N-nitroso-N-methylurea. Quercetin can curtail the levels of IL-6, alpha-fetoprotein, and caspase-3, illustrating its anti-tumor properties. Besides that, quercetin promotes the proliferation of spermatogenic cells while diminishing the RNA levels of Bax and MPO within the testes, improving the testicular cancer in rats stimulated by N-nitroso-N-methylurea.

Peyronie’s disease refers to the fibrotic lesion of the corpora cavernosa tunica albuginea, resulting in the appearance of single or several plaques or indurations located on the dorsal or lateral side of the penis. Wogonin, a compound belonging to the flavone family, has been found to address Peyronie’s disease by preventing cell proliferation, downregulating the secretion of monocyte chemoattractant protein (MCP)-1 in human tunica albuginea cells (79).

Erectile dysfunction significantly diminishes the overall wellbeing of middle-aged men. The findings revealed that isorhamnetin, being a type of flavonol, is capable of restoring erectile function in rats with diabetes, elevating the count of endothelial cells, and improving the morphology of collagen fibers. Simultaneously, isorhamnetin mitigates the damage instigated by high glucose to the endothelial cells of the cavernous body by counteracting ferroptosis and oxidative stress, which contributes to the restoration of erectile function in diabetic rats and improves tissue morphology (80).

#### 2.6.3 Female reproductive system

**FIGURE 7:**
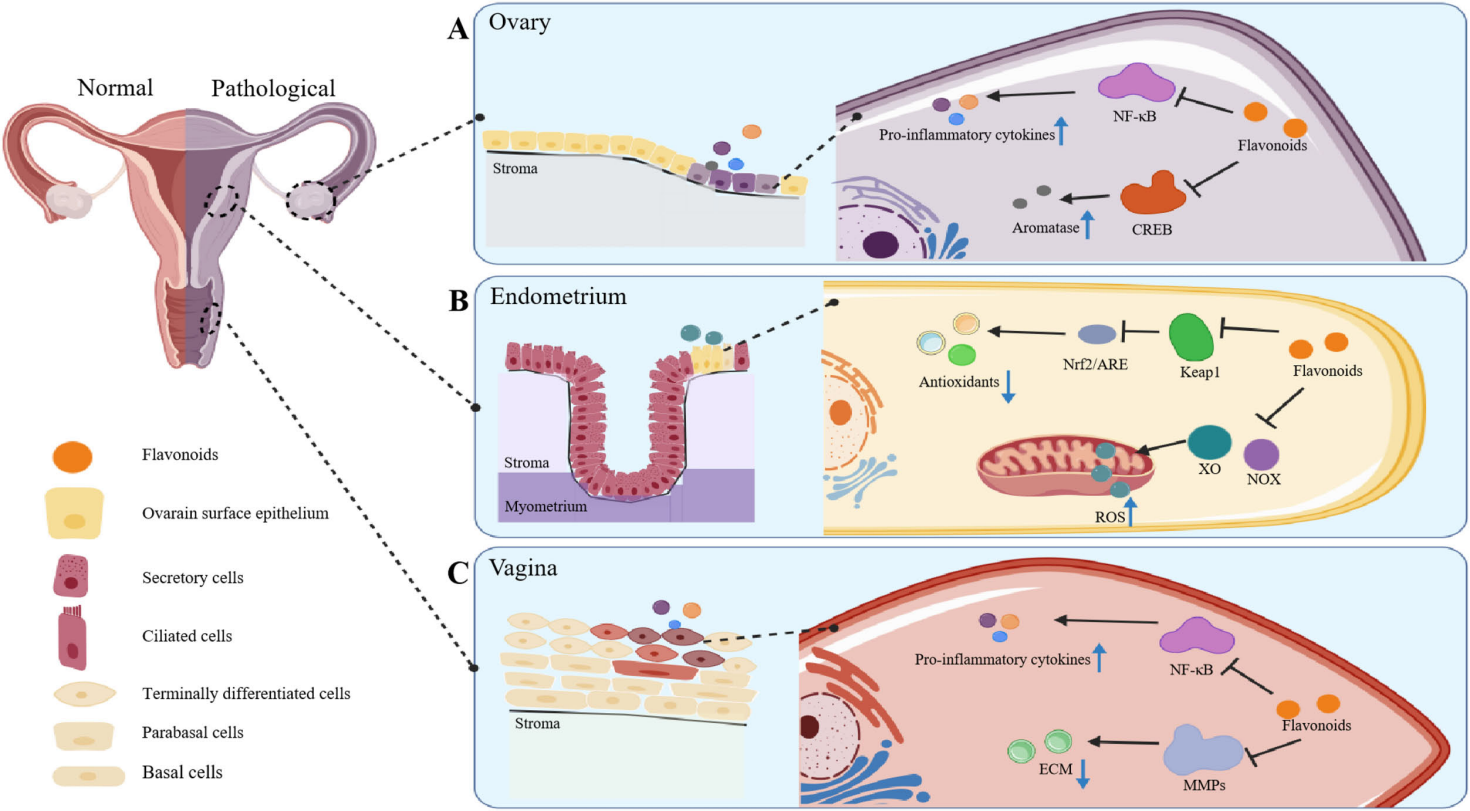
The ways in which flavonoids operate to ameliorate conditions of the female reproductive system. **(a)** In the ovaries, flavonoids curtail the NF-κB pathway to exert anti-inflammatory effects and curb CREB-mediated aromatase expression to modulate hormones, averting polycystic ovary syndrome. **(b)** In the endometrium, flavonoids promote Nrf2 nuclear translocation and block ROS-related enzymes like NOX and XO, combating oxidative stress. **(c)** In the vagina, flavonoids counter the NF-κB pathway and reduce MMPs-2/9 overexpression, relieving ECM degradation and maintaining mucosal integrity via anti-inflammatory effects.

The female reproductive system is essential not only for reproductive functions but also contributes to the regulation of female endocrine through hormone secretion, thus significantly influencing women’s overall health, both physically and mentally (as noted in Figure 7).

##### 2.6.3.1 Breast diseases

For women, malignant tumors like breast cancer are quite widespread, with breast cancer being the second most prevalent cancer form globally and the fifth top contributor to cancer mortality worldwide. Astragalin—a plant-sourced flavonol—lowered the expression of essential enzymes that contribute to the process of glycolysis. Moreover, it curtails glucose uptake and lactate generation. Mechanistically, astragalin exerts its anti-glycolytic and anti-proliferative effects by stimulating AMPK while concurrently inhibiting the mechanistic target of rapamycin signaling pathway. Such findings imply that astragalin may hold promise as a therapeutic option for managing breast cancer (81).

##### 2.6.3.2 Ovarian diseases

Polycystic ovarian syndrome is a metabolic disorder primarily affecting premenopausal women, involving both reproductive and endocrine systems. Formononetin, a primary bioactive isoflavone compound found in Astragalus, effectively reduces the expression of inflammatory factors in the ovarian tissue of dehydroepiandrosterone-induced polycystic ovary syndrome rats. Simultaneously, the administration of formononetin notably decreases ovarian cell apoptosis, elevates the concentration of the anti-apoptotic protein Bcl-2, and lowers the amounts of the apoptotic markers, including Bax and cleaved caspase-3. Additionally, formononetin ameliorates the pathological process of polycystic ovary syndrome by impeding the NLRP3 activation, conferring mitigation of inflammation and cell death (82).

##### 2.6.3.3 Uterine diseases

Endometritis is a reproductive disease, with bacterial pathogens recognized as the primary contributing factor. The study demonstrated that puerarin demonstrated efficacy in addressing endometritis by counteracting reversing the Staphylococcus aureus-triggered increase in ferroptosis and the proteins linked to the P2 × 7 receptor/NLRP3 pathway (83).

Endometrial hyperplasia refers to a non-physiological and non-invasive abnormal proliferation of the endometrium. Chrysin displays its anti-proliferative, antioxidant, and anti-inflammatory properties by reducing the cyclin D1 levels, increasing caspase-3 and Bax content, hence lessening estradiol-induced uterine weight gain and related histopathological alterations, conferring alleviation of endometrial hyperplasia (84).

Endometrial cancer is a prevalent form of gynecological tumor. Recent research focused on the structural customization of the flavonoid framework through organopalladium-mediated C-C bond formation led to the creation of a benzopyran derivative featuring a 4-fluoro phenyl substituent at position 2. This modified structure exhibited remarkable antitumor activity, resulting from the combined suppression of PARP and tubulin, consequently highlighting substantial antiproliferative effects against endometrial carcinoma (85).

##### 2.6.3.4 Cervical diseases

Cervical cancer is regarded as the second most common type of cancer that impacts women. Kuwanon C, a substance classified within the flavone family, has been found to reduce the percentage of HeLa cells in the G1/G0 and G2/M stages of the cell cycle, while increasing the percentage of cells present in the sub G1 phase, hampering the growth of HeLa cells. In furtherance, kuwanon C upregulates the expression of genes associated with apoptosis, including DNA-damage-inducible 45A and caspase-3, and induces a reduction in MMPs, consequently triggering apoptosis in HeLa cells (86).

### 2.7 Endocrine system

**FIGURE 8:**
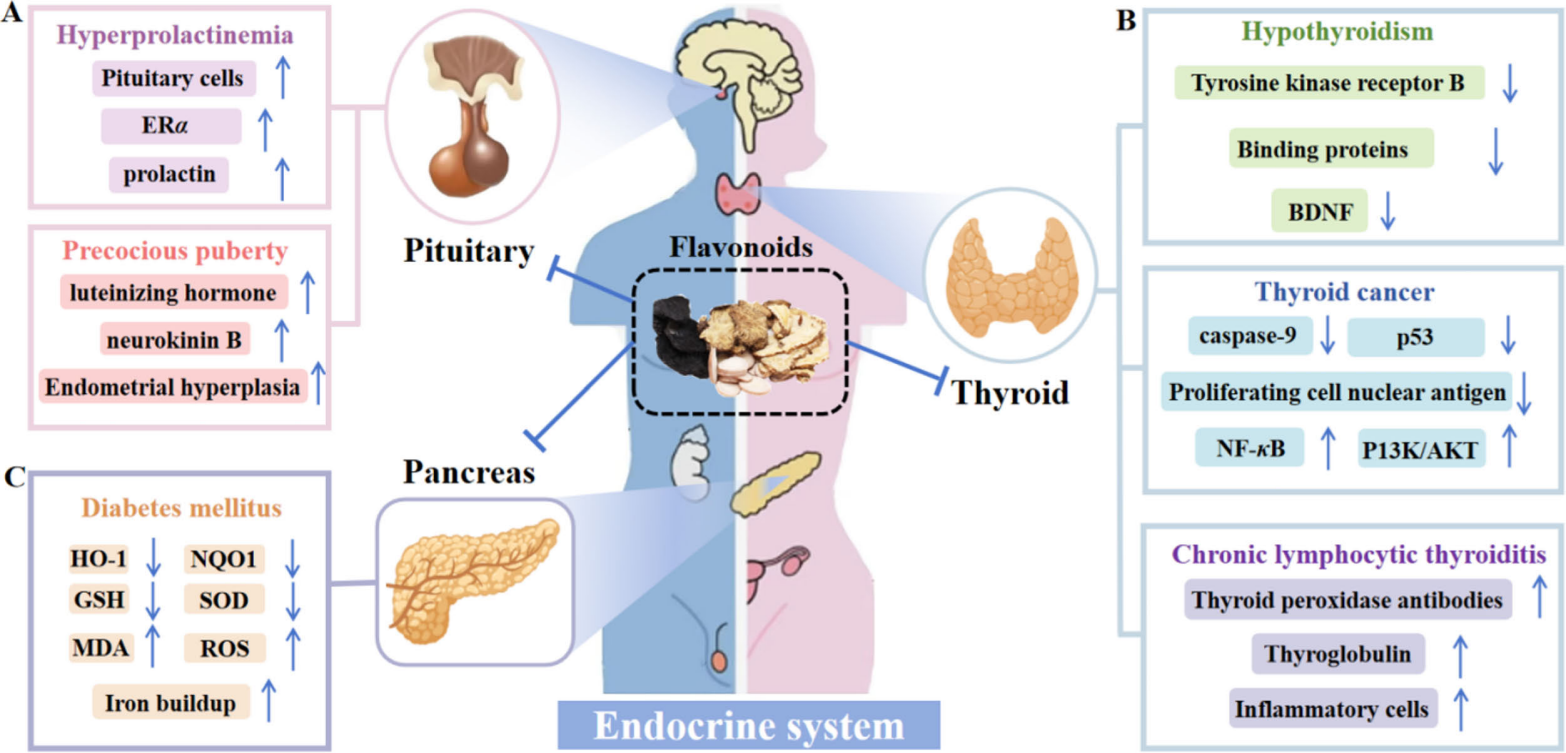
Mechanism of action of flavonoids to ameliorate endocrine system disorders. **(A)** Flavonoids inhibit pituitary cell proliferation, regulate hormone levels, alleviate hyperprolactinemia, and prevent obesity-related precocious puberty. **(B)** In the endometrium, flavonoids promote Nrf2 nuclear translocation and block ROS-related enzymes like NOX and XO, combating oxidative stress. **(C)** In the vagina, flavonoids counter the NF-κB pathway and reduce MMPs-2/9 overexpression, relieving ECM degradation and maintaining mucosal integrity via anti-inflammatory effects.

The endocrine system is responsible for secreting various hormones into the bloodstream. These hormones act on target organs and target cells throughout the body through the transportation of body fluids, precisely regulating important physiological activities such as growth and development, metabolism, and reproduction, and sustaining the homeostasis of the body’s internal environment and functional balance of the organism (as presented in Figure 8).

#### 2.7.1 Pituitary diseases

Hyperprolactinemia is a syndrome of hypothalamic-pituitary axis reproductive endocrine disorders, expressed by elevated serum prolactin levels and its associated clinical manifestations. Casticin is a flavonol compound. Following casticin intervention, there was a significant reduction in both pituitary cell growth and prolactin secretion, with no impact on the growth or prolactin synthesis of normal pituitary cells. These findings indicate that casticin hampers prolactin synthesis by negating the effects of estrogen stimulation, contributing to combating hyperprolactinemia (87).

Precocious puberty is a collection of abnormal pubertal development diseases, mainly manifested by the premature appearance of secondary sexual characteristics in affected individuals. The compound epigallocatechin-3-gallate notably postponed the vaginal opening time in these HFD-induced precocious puberty rats. Concomitantly, it significantly reduced the serum levels of neurokinin B and luteinizing hormone, the ovary’s expression of the neurokinin B protein, and observed the thickness of the endometrium. This results in an elevation in the expression of neurokinin B and its receptor (neurokinin 3), thereby inhibiting obesity-associated precocious puberty (88). As well, epigallocatechin-3-gallate intervention restores the balance of gut microbiota disrupted by HFD by enhancing the population of beneficial microbes like *Akkermansia*. Epigallocatechin-3-gallate also down-regulates the expressions of L-tryptophan, serotonin E2 levels, Cyp1b1 and Col3a1 genes, and reduces the ratio of firmicutes/bacteroidetes, thus addressing precocious puberty (89).

#### 2.7.2 Thyroid diseases

Hypothyroidism is a systemic hypometabolic syndrome instigated by insufficient generation or efficacy of thyroid hormones. The administration of chrysin treatment substantially mitigated different types of cognitive impairments in mice with hypothyroidism, reinstating their cognitive adaptability and elevating the levels of brain-derived neurotrophic factor (BDNF), Tyrosine kinase B, and cyclic adenosine monophosphate (cAMP) response element binding protein (CREB) linked to the BDNF/tropomyosin-related kinase B/CREB signaling pathway (90).

Chronic lymphocytic thyroiditis is a long-lasting condition resulting from the immune response mistakenly targeting the thyroid tissue. Puerarin can hamper the expression of thyroglobulin and thyroid peroxidase antibodies, conferring mitigation of the inflammatory cell infiltration within the tissues. Beyond that, puerarin alleviates chronic lymphocytic thyroiditis by impeding the pyroptosis signaling pathway, so as to suppress macrophage polarization and reduce inflammatory damage (91).

#### 2.7.3 Diabetes

Diabetes mellitus is a long-term metabolic disorder that is primarily divided into three distinct categories: type 1 (manifested by inadequate insulin secretion), type 2 (prompted by resistance to insulin), as well as gestational diabetes (characterized by maternal peripheral insulin resistance). Grape seed proanthocyanidins are anthocyanidin compounds, which induce the interaction between Nrf2 protein and antioxidant response elements, activate the HO-1 and NQO1 expression, exert properties that counter oxidative harm and impede ferroptosis. They also minimize iron accumulation in pancreatic β-cells, and lower lipid peroxidation levels, conferring mitigation of symptoms associated with type 2 diabetes mellitus (92).

### 2.8 Nervous system

**FIGURE 9:**
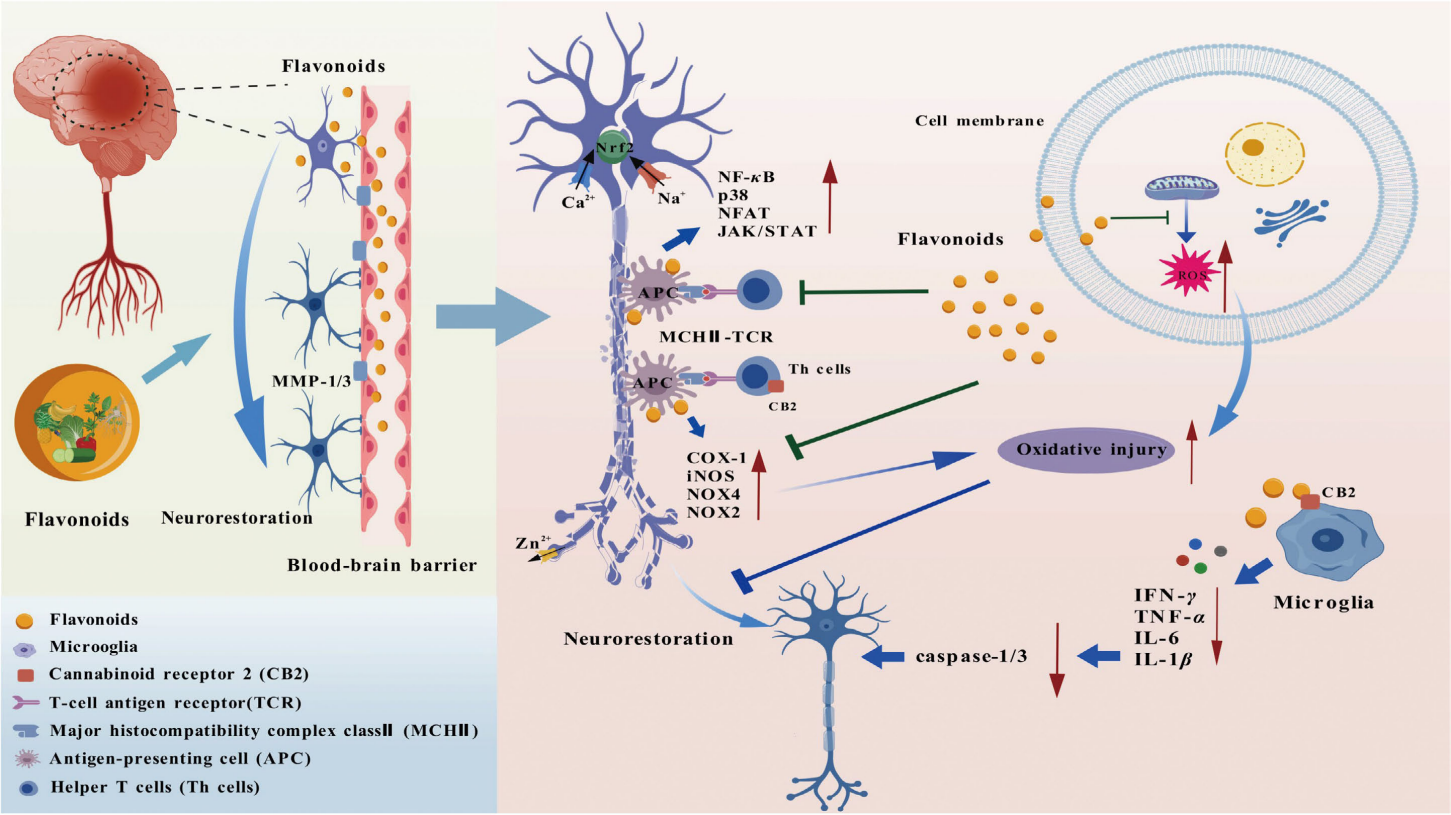
The repair mechanism of flavonoids for nerve injuries.

The nervous system is responsible for receiving, conducting, and processing electrical and chemical signals throughout the body through a complex network of neurons. It precisely regulates physiological activities and behaviors such as sensation, movement, thinking, and memory of the body (as displayed in Figure 9).

#### 2.8.1 Brain diseases

Transient ischemic attack is expressed by temporary neurological dysfunctions arising from local ischemia in the brain or retina. Breviscapine belongs to flavone compounds. It was observed that breviscapine was effective in reducing both cerebral infarction size and the volume of brain water, while inhibiting the levels of inflammatory cytokines in both serum and hippocampal tissue to alleviate inflammatory reactions. Plus, it also reduced the oxidative damage within the hippocampal region, inhibited PARP-1 expression to mitigate apoptosis, and suppressed the activation of microglial activation, alleviating damage inflicted on ischemia/reperfusion in the brain and enhancing neurological performance in rats (93).

Hypoxic-ischemic encephalopathy refers to brain injury resulting from a combination of cerebral ischemia and hypoxia due to diverse factors. The compound quercetin promotes the activity of SIRT1, leading to a decrease in the acetylation of high-mobility group box1 (HMGB1), restricting the translocation between the nucleus and cytoplasm as well as the release of HMGB1. This helps alleviate neuroinflammatory responses, reduce the occurrence of neuronal death, and promote the recovery of neurological functions. Additionally, Chen et al. (94) constructed an oxygen-glucose deprivation model utilizing mouse BV2 microglia was developed to mimic the hypoxic-ischemic conditions found *in vivo*. The application of quercetin was shown to alleviate damage to brain structures, apoptosis, as well as the loss of neurons in the hippocampus.

Intracerebral hemorrhage occurs when blood vessels within the brain rupture without any external injury, resulting in the buildup of blood within the brain tissue. Didymin, a compound classified as dihydroflavone, notably improved neurological deficits following the occurrence of intracerebral hemorrhage, it reduced brain edema and the integrity of the blood-brain barrier (BBB) was preserved. This effect was linked to the upregulation of RAF-kinase inhibitor protein, the reduced levels of microglial pyroptosis-related molecules and inflammatory mediators. Additionally, didymin reduced the presence of microglia that were positive for caspase-1. Mechanistically, didymin binds to apoptosis-associated speck-like protein containing a CARD via RAF-kinase inhibitor protein, blocking the activation and formation of the NLRP3, consequently inhibiting pyroptotic cell death and the neuroinflammatory response arising from the caspase-1/gasdermin D signaling cascade. Thus, didymin might serve as a promising therapeutic option for alleviating both inflammation and damage in the brain following intracerebral hemorrhage (95).

Subarachnoid hemorrhage is an acute cerebrovascular disease characterized by the rupture of blood vessels located at the base or surface of the brain, causing blood to enter the subarachnoid space. Daidzein is a natural isoflavone compound. Results showed that both daidzein and its metabolite equol significantly reduced the rates of intracranial aneurysm formation and the expression of inflammatory cytokines, which was facilitated by the activation of estrogen receptor β (ERβ). Moreover, the shielding impact of daidzein was found to rely on its transformation into equol due to the action of intestinal flora. These results suggest that both daidzein and equol can alleviate the development of intracranial aneurysms and the associated risk of subarachnoid hemorrhage through the regulation of inflammatory processes (96).

Alzheimer’s disease (AD) is a chronic neurodegenerative disorder triggered by multiple factors. Clinically, it presents with a progressive cognitive dysfunction along with behavioral deficits. Neobavaisoflavone is classified as an isoflavone compound. Findings indicated that neobavaisoflavone improved both the exploratory capacity and spatial memory, shortened escape latency, and reduced hippocampal neuronal apoptosis, therefore improving cognitive function and pathological damage in mice with AD (97). Parkinson’s disease (PD), the second most prevalent neurodegenerative disorder following Alzheimer’s, is defined by a progressive decline in motor capabilities. Icaritin, a type of flavonol, has been shown to increase the population of synapse-rich cells, improve the survival of dopaminergic neurons and mitigate neuroinflammation, promote the astrocyte functional recovery, conferring mitigation of PD (98). Europinidin is grouped as an anthocyanidin compound. This compound dose-dependently lessened acetylcholinesterase activity, counteracted the breakdown of acetylcholine, sustained adequate levels of acetylcholine within the synaptic cleft, improved both cognitive abilities and motor skills, conferring alleviation of PD (99).

Demyelination is characterized primarily by myelin loss, typically with a relatively minor impact on the neuronal cell bodies and their axons. Apigenin reduced the severity and relapse rate in mice subjected to experimental autoimmune encephalomyelitis, inhibited the surface expression of molecules on dendritic cell surface and modulated inflammatory pathways, regulated the characteristics of immune cell phenotypes, heightened regulatory T lymphocytes, exerting significant anti-neuroinflammatory properties (100).

Cerebral edema refers to a condition prompted by the excessive buildup of interstitial fluid within brain parenchyma, leading to the swelling of cellular structures or surrounding tissues and subsequently ischemic damage. Dihydroquercetin, a compound belonging to the dihydroflavone class, was shown to enhance the integrity of BBB, and diminish leakage following subarachnoid hemorrhage, conferring mitigation of cerebral edema in rats inflicted by subarachnoid hemorrhage (101).

Intracerebral hematoma denotes a mass formed due to blood accumulation in the brain’s parenchyma, a consequence of ruptured blood vessels. In a mouse model of intracerebral hemorrhage, intervention with wogonin upregulated the expression of anexelekto, the proto-oncogene tyrosine kinase known as MERTK, and CD36 in perilesional microglia through the PPAR-γ signaling pathway, consequently promoting the process of phagocytosis, accelerating the removal of hematomas, and enhancing the recovery of neurological functions. Additionally, wogonin was found to remarkably alleviate the responses associated with inflammation and oxidative stress, fostering an improved internal condition conducive to neural recovery, and indirectly aiding in the management and healing of hematomas (102).

Meningitis refers to infectious inflammation affecting the meninges and the space beneath the arachnoid layer. Quercetin is capable of reducing the levels of pro-inflammatory cytokines and markers associated with BBB permeability, upregulating the expression of angiogenesis-related genes, alleviating parasite-induced tight junction disruption, and inhibiting the activation of the PI3K/Akt/ERK signaling cascade triggered by parasites—thereby conferring therapeutic effects against meningitis (12).

#### 2.8.2 Spinal cord diseases

Spinal cord inflammation is created by assorted infections or allergic reactions that affect either the gray or white matter of the spinal cord. Tangeritin, a compound derived from citrus plants known as a flavone, surprisingly counteracted post-spinal cord injury inflammation. The specific mechanisms involved are associated with the suppression of inflammatory mediators and oxidative stress markers, and promoting the transformation of microglia from the pro-inflammatory M1 condition to the anti-inflammatory M2 state through the heightening of the Sestrin 2/Keap1/Nrf2 signaling cascade, introducing a promising therapeutic intervention for the management of spinal cord injury (103).

Compressive myelopathy encompasses a series of neurological dysfunctions driven by spinal cord compression. Hydroxysafflor yellow A, a chalcone analog, attenuated oxidative stress through lower MDA levels, MPO and NO concentrations while enhancing SOD activity. Suppressing the inflammation through obstructing NF-κB activation. Concomitantly, it protects neurons from apoptosis, resulting in fewer TUNEL-positive cells, contributing to enhance the restoration of limb functionality following spinal cord compression injuries (104).

#### 2.8.3 Disorders of peripheral nervous system

Trigeminal neuralgia is a medical condition defined by brief, paroxysmal severe pain, occurring without accompanying neurological deficits. Anthocyanins are components of the anthocyanidin class within the flavonoid compounds. Anthocyanidins have the ability to hamper neuroinflammation through the reduction of pro-inflammatory cytokine production, enhance autophagy, and diminish the infiltration of immune cells, consequently delaying and reducing the severity of trigeminal neuralgia. Additionally, it has the potential to improve motor function and prevent recurrence, introducing novel potential avenues for addressing trigeminal neuralgia (105).

### 2.9 Cutaneous system

**FIGURE 10:**
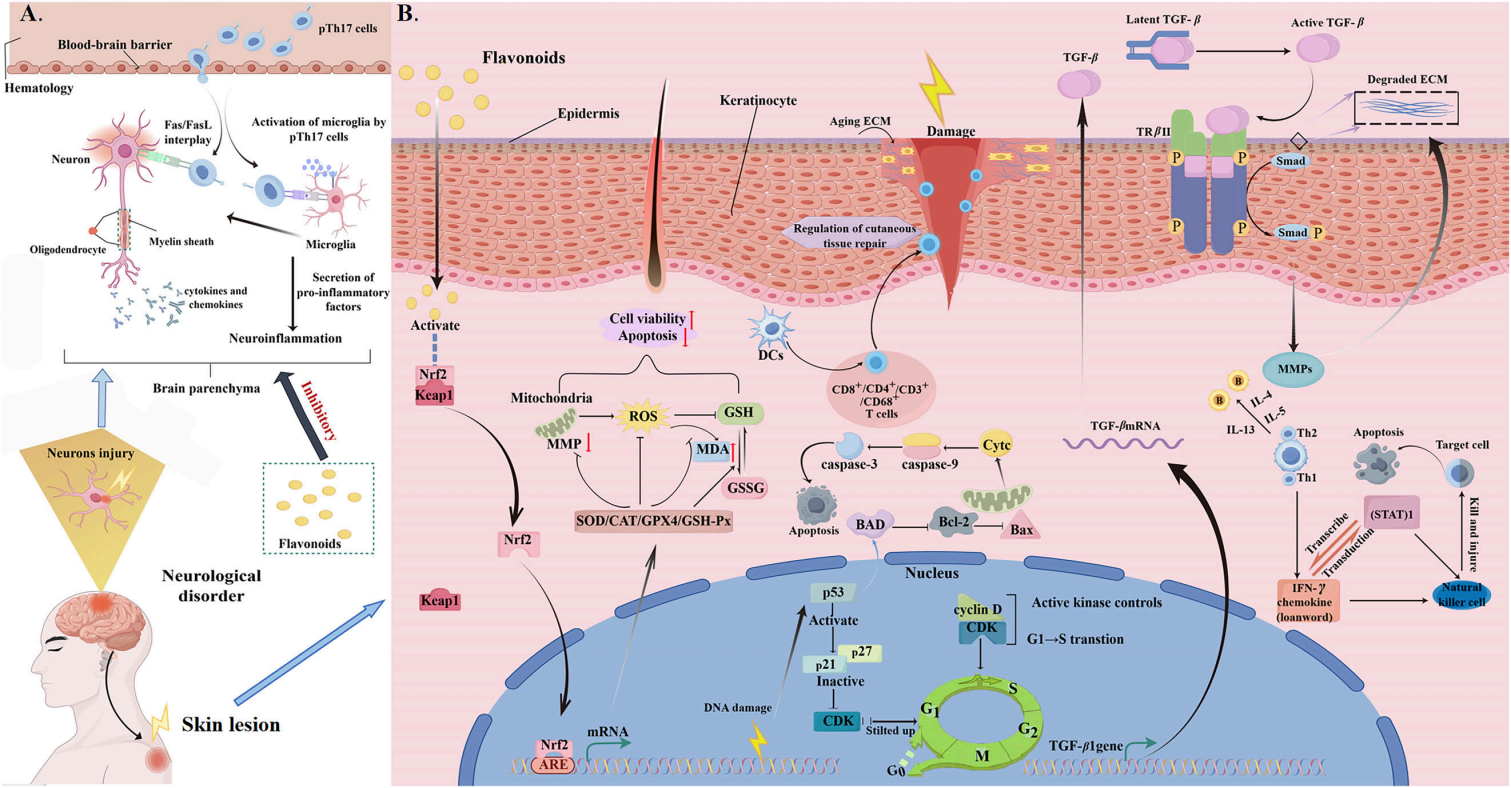
Mechanisms of action of flavonoids in improving skin damage. **(A)** Inhibition of neuroinflammation by flavonoids following brain injury. Flavonoids attenuate brain injury-induced neuroinflammation (and alleviate related neurocutaneous disorders) by inhibiting pathogenic Th17 cell activation (to reduce microglia activation and pro-inflammatory cytokines) and modulating Fas/FasL to decrease neuronal apoptosis. ** (B)** Mechanisms of flavonoids in repairing skin damage. Flavonoids repair skin damage via multiple pathways: activating Nrf2 to boost antioxidants and reduce ROS, inhibiting apoptosis (via caspase-3/9) and cell cycle (via cyclin D/CDK), curbing inflammation, and activating TGF-β to promote fibroblast proliferation for tissue repair.

The cutaneous system covers the surface of the human body, distributing a wealth of nerve endings and receptors, and these nerves are closely connected to the nervous system, the touch, pain, temperature and other information are quickly transmitted to the central nervous system, so that the human body can promptly perceive and respond to external stimuli (as summarized in Figure 10).

A sunburn is a kind of traumatic skin reaction created by the skin’s exposure to intense light. Isosakuranetin belongs to the class of flavanones. Equol, an isoflavanone compound, significantly reduced the formation of cyclobutane pyrimidine dimers simulated by ultraviolet radiation exposure. It was noted that the rate of dimers’ clearance did not experience a noteworthy acceleration (106). Vicenin-2, a flavone, significantly improved the photoaging manifestations such as skin moisture loss, heightened tenderness, hyperpigmentation, and deepened wrinkles in ultraviolet-irradiated mouse skin. Moreover, introduction of vicenin-2 performed shielding impact against photo-aging benefits by impeding the GSK-3β expression and phosphorylation, alongside throttling the PI3K/Akt signaling pathway, thus contributing to hamper inflammation and apoptosis (107).

Atopic dermatitis is a persistent inflammatory condition of the skin, marked by recurrent eczema along with inflamed skin lesions, impaired barrier function, and severe pruritus. Nepetin, classified as a flavone compound, led to a notable decrease in the levels of cytokines associated with inflammation, skin damage, and behavioral metrics in a mouse model of atopic dermatitis triggered by 1-chloro-2,4-dinitrobenzenes (108).

Sjogren’s syndrome is prompted by the generation of autoantibodies which leads to the inflammatory response, swelling, as well as harm to the glands that secrete fluids, chiefly affecting the salivary and tear-producing glands. In a mouse model, which spontaneously mimics Sjogren’s syndrome and triggers inflammation in the salivary gland, quercetin has the ability to distinctly reduce the loss in salivary flow, salivary gland injury apoptosis, as well as immune response observed in mice, and also decrease the leptin level in the serum. Regarding the modulation of signal transduction, quercetin hinders the associated JAK2/STAT3 signaling, therefore safeguarding salivary gland epithelial cells from apoptosis, and thus sufficiently mitigating the damage to the salivary glands influenced by Sjogren’s syndrome (109).

Psoriasis is a long-lasting autoimmune disorder that affects the skin. This condition arises from an immune system malfunction, it leads to an accelerated and uncontrolled generation of skin cells, which is instigated by the atypical proliferation of epidermal keratinocytes along with the infiltration of immune cells. The introduction of dihydromyricetin has curtailed the skewing of macrophages toward the inflammatory M1 type by impeding the TLR4/NF-κB axis. Concretely, this in turn negated the development of subsets of helper T cells, specifically Th1 and Th17 cells, conferring remission of the immunoinflammatory response associated with psoriasis (110).

Ichthyosis is a range of hereditary keratinization-disordered skin diseases, predominantly expressed as dry skin with fish-scale-like desquamation. Delphinidin is classified under the anthocyanidin compounds. Both in submerged *in vitro* cultures and in three-dimensional epidermal equivalent systems, delphinidin can enhance the differentiation of keratinocytes, boost the promoter activity of human involucrin, as well as increase the transcription and translation of epidermal differentiation markers. Additionally, delphinidin has the capacity to promote keratinization by enhancing the expression of key proteins linked to the keratinization process. Hence, delphinidin may fortify the epidermal barrier by promoting epidermal differentiation and strengthening the keratinization process, thus exerting beneficial effects for conditions like ichthyosis. Exceptionally, when normal human epidermal keratinocytes are treated with delphinidin, there is a minimal reduction in the cell viability, and it does not trigger apoptosis, suggesting a favorable safety profile (111).

Purpura is a frequent condition characterized by color alterations after hemorrhage in the skin and mucous surfaces. The clinical signs consist of petechiae and ecchymoses visible on the skin, which do not fade even when being pressed. After the intervention of proanthocyanidins, it hampered the TLR4/myeloid differentiation protein 88 (MyD88)/NF-κB pathway, and then reduced the infiltration of inflammatory cells and the deposition of IgA in the mesangial area of the kidney. Then, it reduced the biomarker for oxidative damage MDA, increased the levels of SOD and CAT, alleviated the oxidative damage, demonstrating a promising potential approach for managing Henoch-Schönlein purpura (112).

Malignant melanoma originates from melanocytes and is the most dangerous form of skin cancer. The flavones xanthomicrol and eupatilin demonstrated antitumor activities in the malignant melanoma cell lines by lessening cell growth, inducing apoptosis, altering cell morphology, regulating lipid metabolism, boosting the production of ROS, as well as reducing the MMP (113).

### 2.10 Other diseases

Flavonoids have demonstrated substantial potential in the field of anti-addiction and addiction regulation, offering a novel direction for natural therapeutic approaches in the prevention and intervention of addictive disorders. Extensive research has confirmed that these compounds exert their effects primarily through two core pathways: the precise regulation of the reward circuitry in the central nervous system (CNS) and the modulation of addiction-related behaviors. Their characteristic of multi-target synergy can simultaneously alleviate the pathological damage associated with addiction, further enhancing the intervention efficacy (114). The imbalance of the CNS reward circuitry constitutes a key pathological basis for the development of addiction (115). Flavonoids achieve balanced regulation of the reward circuitry mainly by targeting the dopaminergic system (116), γ-aminobutyric acid (GABA) ergic system (117), and glutamatergic system (118), while integrating other auxiliary pathways. Dopamine is a crucial neurotransmitter in the reward circuitry. Addictive substances (e.g., cocaine) often promote the excessive release of dopamine in the nucleus accumbens (NAc, a core brain region of the reward circuitry), thereby potentiating the “reward effect”and triggering substance craving (119). Flavonoids such as quercetin can inhibit the activity of the dopamine transporter (DAT) to reduce the reuptake of dopamine in the synaptic cleft (120), consequently attenuating the abnormal NAc dopamine release induced by addictive substances and fundamentally weakening the substance dependence tendency caused by the “reward effect.” The weakened inhibitory function of GABAergic neurons (121) and abnormal glutamatergic projections (122) are important contributors to anxiety, tremors, and cognitive control deficits (e.g., impulsive drug use) during addiction withdrawal. Flavonoids such as baicalin (123) can enhance the inhibitory effect of GABAergic neurons and suppress the overactivation of noradrenergic neurons in the locus coeruleus, thereby alleviating symptoms such as anxiety and tremors during the withdrawal period of opioid and alcohol addiction. Beyond the aforementioned core systems, flavonoids also assist in regulating the reward circuitry through two common mechanisms (124): first, reducing NO levels to mitigate neuroexcitotoxicity and prevent reward circuitry damage caused by excessive excitation; second, antagonizing calcium channels to inhibit calcium ion influx and maintain the electrophysiological homeostasis of neurons. Additionally, the antioxidant properties of certain flavonoids (e.g., fisetin) exert neuroprotective effects, reducing neuronal damage induced by oxidative stress during addiction and supporting the functional recovery of the reward circuitry (116).

Based on the precise regulation of the reward circuit, flavonoids exhibit clear intervention effects on behaviors induced by alcohol, nicotine, opioids, and other addictive substances (such as heroin and methamphetamine), with different flavonoids showing specific focus in their effects on particular addictive substances (124). For alcohol addiction, multiple flavonoids intervene through different pathways, and their effects are synergistic: resveratrol can inhibit the formation of ethanol-induced conditioned place preference (CPP), daily administration can shorten the extinction period of alcohol-induced CPP and also inhibit the reinstatement behavior triggered by low-dose ethanol. Meanwhile, when resveratrol at various doses was combined with ethanol, it did not affect the spontaneous activity or motor coordination of mice, ruling out interference with basic behaviors. Moreover, its overall intervention effect is similar to that of acamprosate, a clinical anti-alcohol addiction drug, suggesting that resveratrol has potential application value in the prevention and intervention of ethanol addiction (114). Quercetin and myricetin can inhibit the acquisition of ethanol-induced CPP by activating GABAA receptors and peroxisome proliferator-activated receptors (PPARs), inhibiting the glutamatergic system, and reducing NO levels. Daily administration can accelerate CPP extinction and effectively block the reinstatement induced by low-dose ethanol (118, 125). The core mechanism of nicotine addiction is the activation of α4β2 nicotinic acetylcholine receptors (nAChR) in the brain, which promotes dopamine release to produce a reward effect (126). Flavonoids mainly exert their effects by blocking these receptors or regulating the downstream reward circuit: The anti-nicotine addiction effect of resveratrol is similar to that of varenicline (a partial agonist of α4β2 nAChRs), a clinical smoking cessation drug. It cannot only significantly inhibit the formation of nicotine-induced CPP but also block the reinstatement triggered by low-dose nicotine. Its mechanism involves multi-target synergy: inhibiting N-methyl-D-aspartate receptors, enhancing GABAergic neurotransmission, activating PPARs, reducing nitric oxide synthase activity, and antagonizing calcium channels. Additionally, the combination of nicotine and resveratrol does not affect the motor coordination of mice, verifying its safety (117). For opioid addiction, epigallocatechin gallate (EGCG) can alleviate withdrawal symptoms by inhibiting the rebound increase in cyclic adenosine monophosphate (cAMP) levels in the locus coeruleus, and correct dopamine system disorders and alleviate psychological dependence by inhibiting dopamine D2 receptor signaling, thereby exerting a potential intervention effect on opioid addiction (127). For heroin addiction, resveratrol and quercetin can enhance the inhibitory postsynaptic currents mediated by GABAA and GABAB receptors in dopamine neurons of the ventral tegmental area, and also increase the level of IL-6 in the striatum, thereby effectively alleviating heroin withdrawal symptoms and preventing the formation of heroin-induced CPP (124). For methamphetamine addiction, flavonoids exhibit potential effects in multiple aspects (124): Quercetin can alleviate methamphetamine-induced anxiety-like behaviors and improve mitochondrial dysfunction and neuroinflammation; resveratrol can reduce methamphetamine-induced dopamine release and hyperlocomotion; in addition, genistein affects addiction-related neural adaptation by regulating estrogen receptors and the AMP-activated protein kinase (AMPK) signaling pathway, further expanding the application scope of flavonoids in anti-addiction.

The anti-addictive effects of flavonoids do not rely on a single target, but rather achieve comprehensive intervention in the addiction process through multi-dimensional synergy, including “regulating the reward circuitry (dopaminergic/GABAergic/glutamatergic systems), improving substance-related behaviors (reducing craving, alleviating withdrawal symptoms, inhibiting relapse), and mitigating pathological damage (antioxidation, anti-inflammation).” From a pharmacological perspective, the anti-addictive effects of flavonoids further enrich our understanding of their pharmacology. These natural compounds not only excel in anti-inflammatory and antioxidant activities but also can precisely target central addiction-related pathways, providing new natural intervention options for the prevention and management of addictive disorders involving alcohol, nicotine, opioids, and other substances. In-depth exploration of their mechanisms of action in the future is expected to promote the clinical translation of natural products in the field of addiction treatment.

## 3 Conclusion and perspectives

The efficacy of traditional Chinese medicine (TCM) has been verified by hundreds of years of extensive clinical applications. Flavonoids, as polyphenolic secondary metabolites widely found in TCM and plants, are an important bridge between TCM and modern medicine. Flavonoids work synergistically through multi-targets and multi-pathways, and their core mechanisms cover antioxidant, anti-inflammatory, apoptosis and proliferation, metabolic signaling pathways and anti-addiction. However, flavonoids are still facing challenges in terms of production and resource security, bioavailability, targeting and clinical translation. The production and preparation of flavonoids depend on limited plant resources, and the traditional extraction and purification process is complex, not only easy to destroy the structure of the active ingredients, but also subject to the long growth cycle of the plant, the geographical environment and other limitations, resulting in unstable yield and high cost (128). The quality of resources lacks uniform standards, and the market has problems such as uneven quality of raw materials and fluctuations in the content of active ingredients, which affects its pharmacological effect and stability of clinical application. To address these limitations, at the production and preparation level, on the one hand, chemical synthesis technology can be relied on to build the mother nucleus and derivatives of flavonoids with basic chemical raw materials, to get rid of the dependence on plant resources, and at the same time, integrate green synthesis and high-efficiency catalytic system to improve the synthetic efficiency and reduce the cost of the environment (129). On the other hand, microbial cell factories or enzyme-catalyzed biosynthesis systems can be developed to achieve the direct production of flavonoids *in vitro*, simplify the extraction and separation steps, and shorten the production cycle (130), and can also be combined with new environmentally friendly separation techniques, such as supercritical fluid extraction and high-speed counter-current chromatography, to improve the purity of the target compounds in the extracts. At the level of resource quality assurance, high-quality genes can be screened through genome analysis, molecular breeding can be strengthened to cultivate high-yielding varieties, while the germplasm database can be established to achieve the traceability of raw materials, and the production and marketing standardization can be combined to regulate the production process. In addition, with the help of fingerprinting and multi-indicator quantification (131), metabolomics and process monitoring (132) and industrialization technology integration (133), we can build a whole-chain quality control system to solve the problem of market disruption and to ensure the stable quality of flavonoids from raw materials to products.

Low bioavailability is the core factor restricting the clinical application of flavonoids, and its influencing factors can be divided into the obstacles encountered in the pre-absorption, absorption process, and post-absorption. Pre-absorption obstacles directly affect whether the drug can reach the intestinal absorption site, including water solubility, stability and other aspects. Most flavonoids have low polarity and poor solubility in aqueous environments (e.g., gastrointestinal tract). To address this problem, on the one hand, flavonoids can be modified by means of molecular structure modification, such as salt formation, glycosylation modification, pre-drug modification, or introduction of polar functional groups, to directly enhance water solubility (134–136). On the other hand, flavonoids can be biotransformed using microorganisms or enzymes to generate derivatives with higher water solubility (137). Preparation means can also be used, such as constructing a nano-delivery system to improve the water solubility of flavonoids with the help of nanotechnology, or allowing flavonoids to form eutectic with eutectic formers through non-covalent bonding to change the crystal structure and enhance the water solubility (136). In addition, the process and extraction method can be optimized. Taking baicalin as an example, which is a major active ingredient present in Scutellaria baicalensis, the magnesium baicalin prepared by optimizing the extraction process in the early stage of our work was able to substantially improve the water solubility of baicalin, and subsequent studies have demonstrated that the magnesium baicalin is capable of resisting skin aging (138), antidepressant (139), treatment of non-alcoholic steatohepatitis (140), acute lung injury (141), and ulcerative colitis (142), which is superior to equimolar baicalin. The discovery of magnesium baicalin integrates the traditional theory of traditional Chinese medicine with modern science and technology, overcoming the problem of poor water solubility in the application of baicalin with better therapeutic efficacy, and laying the foundation for the further development and application of the active ingredients of other Chinese medicines. In addition, natural flavonoids are susceptible to degradation by light, alkaline environment and metal ions, and are easily destroyed by gastric acid and digestive enzymes in the gastrointestinal tract, resulting in a decrease in the amount of active drug reaching the intestinal absorption site. In this regard, covalent cross-linking technology can be used to combine flavonoids with cellulose sponges to maintain stable antimicrobial properties during recycling, and also to provide a basic guarantee for the stability of natural flavonoids by avoiding light, alkaline environments, and storage conditions isolated from iron ions (143).

Obstacles in the absorption process determine whether the drug that reaches the target site can effectively enter the blood circulation, in which the obstacles in the penetration of the biological barrier due to poor lipid solubility and high molecular weight of the compounds are important. To address this problem, the structural modification of flavonoids can be carried out, such as combining quercetin with unsaturated fatty acids such as linoleic acid and oleic acid in an esterification reaction to generate quercetin-linoleic acid ester and quercetin-oleic acid ester, to enhance the lipophilicity of quercetin and to improve its skin penetration (144), and this strategy provides a basis for the application of flavonoids in the fields of skin whitening cosmetics and antipigmentation pharmaceutical research and development. Alternatively, adriamycin-loaded bovine serum albumin nanoparticles can be prepared by desolventisation, and then mPEG2000 can be grafted onto the surface of the particles by amide bonding to form new particles, and the PEG hydration layer can be used to reduce the adsorption of plasma proteins in order to prolong the *in vivo* circulation time. Afterward, by the electrostatic effect of positively charged lactoferrin (Lf) and negatively charged new particles under physiological pH, Lf was adsorbed onto the surface of the particles to form dual-modified nanoparticles, and then Lf was specifically bound to the LRP receptor, which was expressed on the surface of endothelial cells of the blood-brain barrier and glioma cells, and triggered the cytotropy and transcytotropy mediated by the receptor, so as to achieve the penetration of the blood-brain barrier and targeting of the glioma cells, and ultimately to make adriamycin accumulate efficiently in glioma regions in the brain. Glioma region in the brain, while reducing the distribution of the drug in normal organs, enhancing the killing effect on glioma cells and reducing off-target toxicity. And solving the problem that traditional chemotherapeutic drugs are difficult to penetrate the blood-brain barrier (145). In addition, flavonoids need to bind to target enzymes (e.g., tyrosinase) *in vivo* in order to play a role, whereas the sugar groups of glycoside derivatives have spatial site-blocking, which will prevent the compounds from approaching the enzyme activity center (e.g., histidine His-85 and aspartic acid Asp-348 residues of mushroom tyrosinase) (144, 146). In this regard, at the level of structure optimization and design, the three-dimensional interaction model of flavonoids and tyrosinase (e.g., mushroom tyrosinase PDB ID: 2Y9X) can be constructed with the help of molecular docking, molecular dynamics simulation, and other Silico techniques, so as to accurately analyze the binding mode of the two and clarify the key amino acid residues of the active center of the enzyme (e.g., histidine His-85, aspartic acid Asp-348) and the interaction sites with the compounds, and thus to identify the interaction sites with the compounds, and thus to identify the interaction sites of the enzyme. This strategy can help to optimize the compound structure, reduce the obstruction of the “compound-enzyme” binding process caused by the spatial resistance of the sugar group, improve the binding efficiency, reduce the proportion of compounds that are metabolically cleared due to the failure of the compounds to act on the target site, improve the stability, and indirectly enhance the bioavailability (146–148). This strategy has great potential for application in tyrosinase-related diseases (e.g., skin pigmentation abnormalities, metabolic disorders, etc.).

Loss of metabolism after absorption further reduces the effective concentration of the drug in the blood. After entering the body, flavonoids are susceptible to inactivation by intestinal UDP-Glycosyltransferase (UGT)-mediated glycosylation reaction, resulting in a shortened retention time of the prodrugs in the bloodstream, while some flavonoids are susceptible to rapid excretion by the kidneys or phagocytosis by the reticuloendothelial system (RES) due to their lack of targeting. To address this problem, the parent nucleus or side chain groups of flavonoids can be modified by chemical means such as methylation, acetylation and glycosidation, e.g., acetylation of the 7-position hydroxyl group of quercetin, to enhance the lipid solubility and membrane permeability, to reduce the inactivation of intestinal UGT-mediated metabolism, to prolong the retention time of the prodrug in the intestinal tract (149, 150), and to reduce the metabolic loss, thus improving the amount and efficiency of the drug into the circulation and thus increasing the bioavailability of the drug. This in turn improves bioavailability. The innovation can also be made at the level of delivery system, based on the principle of “polyphenol-nano-enzyme-probiotics synergistic effect,” using green coating technology to build a composite carrier, for example, through the synthesis of chitosan-modified epigallocatechin gallate (EGCG-CS) capturing gold nano-enzymes to form an ECA complex, and then coating *E. coli* Nissle 1917 (Ecobacteria) and *E. coli* Nissle 1917 (Ecobacteria) to form an ECA complex, which can be used as the delivery system. Nissle 1917 (EcN) to produce ECA@EcN, preserving the activity of each component through phenol-mediated interfacial and electrostatic interactions, as well as enhancing the probiotic resistance to gastrointestinal stress and intestinal retention, and ultimately enhancing bioavailability and IBD therapeutic efficacy through the scavenging of ROS and regulation of intestinal flora (151).

Flavonoids are insufficiently targeted in the *in vivo* delivery process, and it is difficult to accurately enrich in the lesion site, which not only reduces the therapeutic effect, but also may increase the risk of toxicity in non-diseased tissues due to the systemic wide distribution. For example, genistein binds to the estrogen receptor, which may interfere with the endocrine system and is detrimental to adolescent females (152). To address this problem, a star-shaped crosslinked nanocarrier can be constructed with polyglutamic acid, coupled with Angiopep-2 ligand loaded with genistein, which can not only cross the blood-brain barrier through the binding of the receptor but also reduce the aggregation of β-amyloid, which can enhance the targeting and at the same time reduce the toxicity and improve cognitive function (153). On the other hand, multi-targeting systems can be developed with the help of innovative carrier materials, such as Kae-Fe self-assembled nanoparticles, which are laser-activated to target breast cancer foci (154). Targeted delivery of quercetin to osteoarthritic articular cartilage sites can also be achieved with the help of MXene nanosheet carriers (155). In terms of blood-brain barrier, BMSe@BSA was constructed with BSA-stabilized selenium nanoparticles as a carrier to penetrate the blood-brain barrier to target brain lesions in Alzheimer’s disease mice (156).

A variety of flavonoid-rich drugs have been approved and marketed, such as Lianhua Qingdian capsule (157), Compound Danshen tablets (158), Compound Danshen drip pills (159), and Qianlie Shutong capsule (160), and there are also quercetin [Identifier: NCT04907253 (161)], genistein flavonoids [Identifier: NCT01982578 (162)], and anthocyanin supplements [Identifier: NCT02317211 (163)] and other flavonoids are also in the clinical research stage, but flavonoids still face challenges in clinical translation. On the one hand, there is a lack of depth of knowledge about toxic reactions and side effects induced by high doses of flavonoids, a lack of safe dosage recommendations, and the risk of drug combinations (164). Although most flavonoids are considered safe, the public tends to overlook their toxicity and excessive intake, with potential toxicity including carcinogenicity, hepatotoxicity, nephrotoxicity, thyrotoxicity, reproductive effects, effects on intestinal flora and neurobehavioral abnormalities (165). In this regard, cryo-electron microscopy, molecular simulation and structural analysis techniques (166) can be used to explore their targets and toxicological mechanisms to improve the toxicological evaluation system. On the other hand, most of the current studies on flavonoids focus on cell and animal experiments, and there is a lack of cross-species dose conversion (165), and in the future, we can determine the effective dose in humans by constructing a physiologically based pharmacokinetic (PBPK) model (167), carrying out long-term human studies and dose-escalation experiments, and focusing on the safety of long-term use. Flavonoids can also exert synergistic effects in combination with clinical drugs, such as lignans and low-dose paclitaxel synergistically inhibiting esophageal cancer cell migration (168), quercetin synergistically increasing the drug sensitivity of prostate cancer cells with doxorubicin (169), apigenin synergistically enhancing the antiplatelet effect with aspirin (170). And albiziain enhancing the chemotherapeutic effect of 5-fluorouracil on gastric cancer cells (171). However, there are some risks of combination, such as some flavonoids inhibit CYP3A4 enzyme *in vitro* to affect drug metabolism (172), and reduce P-glycoprotein-mediated drug efflux (173), in this regard, the interactions between the compounds and the drugs should be confirmed before using the drugs, and the guidelines for combination should be formulated and improved, and the treatment protocols can be customized through genetic testing (174), in order to reduce the adverse effects and improve the prognosis of the patients.

Future research on flavonoids can break through the existing limitations around the three major directions of “mechanism innovation mining, technology cross-border integration, and application scenario expansion:” in the field of production preparation and resource guarantee, “AI-driven microbial metabolic engineering” can be explored, and through machine learning to predict the regulatory nodes of the key pathway of flavonoid synthesis, targeted transformation of engineering strains to enhance the yield of specific flavonoid derivatives, while developing microfluidic chip-based miniaturized extraction devices to achieve low-energy, high-purity flavonoids rapid isolation, to solve the contradiction between the scale of the traditional process and refinement. In the field of bioavailability, the dynamic binding mechanism between flavonoids and intestinal transporters can be studied in depth, and the three-dimensional structure of the complex can be analyzed to guide the design of flavonoid analogs that can efficiently bind the transporters, and at the same time, the “mucosal adhesion nanogel” can be developed, which can prolong the retention time of the drug through specific interactions with the intestinal mucus layer and break through the absorption simultaneously. We will also develop “mucosal adhesion nanogels” to extend the retention time of the drug through specific interaction with the intestinal mucus layer, and simultaneously break through the barrier restriction and metabolic loss during absorption. In the field of targeting, we will develop delivery systems with special properties (e.g., photothermal conversion, dual-response release) to meet the therapeutic needs of superficial tumors, osteoarthritis and other diseases. In the field of clinical translation, based on precision medicine, individualized dose prediction models can be constructed through genetic testing and machine learning, precise combination solutions of flavonoids and other drugs can be developed for disease subtypes, and guidelines for special populations can be established. Finally, we can expand the application of flavonoids in emerging diseases such as Parkinson’s disease, systemic lupus erythematosus, and novel viral infections, and analyze the targets and combination therapy strategies, so as to provide new candidates for the treatment of related diseases.
